# Identification and Characterization of Human Norovirus NTPase Regions Required for Lipid Droplet Localization, Cellular Apoptosis, and Interaction with the Viral P22 Protein

**DOI:** 10.1128/spectrum.00422-21

**Published:** 2021-08-25

**Authors:** Ju-Bei Yen, Lee-Wen Chen, Ling-Huei Wei, Chien-Hui Hung, Shie-Shan Wang, Chun-Liang Lin, Pey-Jium Chang

**Affiliations:** a Graduate Institute of Clinical Medical Sciences, College of Medicine, Chang-Gung University, Taoyuan, Taiwan; b Department of Pediatrics, Chang-Gung Memorial Hospital, Chiayi, Taiwan; c Department of Respiratory Care, Chang-Gung University of Science and Technology, Chiayi, Taiwan; d Department of Pediatric Surgery, Chang-Gung Memorial Hospital, Chiayi, Taiwan; e School of Medicine, Chang-Gung University, Taoyuan, Taiwan; f Department of Nephrology, Chang-Gung Memorial Hospital, Chiayi, Taiwan; U.S. Food and Drug Administration

**Keywords:** norovirus, NTPase, lipid droplets, apoptosis, P22

## Abstract

The human norovirus (HuNV)-encoded nucleoside-triphosphatase (NTPase) is a multifunctional protein critically involved in viral replication and pathogenesis. Previously, we have shown that the viral NTPase is capable of forming vesicle clusters in cells, interacting with other viral proteins such as P22, and promoting cellular apoptosis. Herein, we demonstrate that NTPase-associated vesicle clusters correspond to lipid droplets (LDs) wrapped by the viral protein and show that NTPase-induced apoptosis is mediated through both caspase-8- and caspase-9-dependent pathways. Deletion analysis revealed that the N-terminal 179-amino-acid (aa) region of NTPase encompasses two LD-targeting motifs (designated LTM-1 and LTM-2), two apoptosis-inducing motifs, and multiple regulatory regions. Interestingly, the identified LTM-1 and LTM-2, which are located from aa 1 to 50 and from aa 51 to 90, respectively, overlap with the two apoptosis-inducing motifs. Although there was no positive correlation between the extent of LD localization and the degree of cellular apoptosis for NTPase mutants, we noticed that mutant proteins defective in LD-targeting ability could not induce cellular apoptosis. In addition to LD targeting, the amphipathic LTM-1 and LTM-2 motifs could have the potential to direct fusion proteins to the endoplasmic reticulum (ER). Furthermore, we found that the LTM-1 motif is a P22-interacting motif. However, P22 functionally augmented the proapoptotic activity of the LTM-2 fusion protein but not the LTM-1 fusion protein. Overall, our findings propose that NTPase may participate in multiple cellular processes through binding to LDs or to the ER via its N-terminal amphipathic helix motifs.

**IMPORTANCE** Human noroviruses (HuNVs) are the major agent of global gastroenteritis outbreaks. However, due to the lack of an efficient cell culture system for HuNV propagation, functions of the viral-encoded proteins in host cells are still poorly understood. In the current study, we present that the viral NTPase is a lipid droplet (LD)-associated protein, and we identify two LD-targeting motifs, LTM-1 and LTM-2, in its N-terminal domain. In particular, the identified LTM-1 and LTM-2 motifs, which contain a hydrophobic region and an amphipathic helix, are also capable of delivering the fusion protein to the endoplasmic reticulum (ER), promoting cellular apoptosis, and physically or functionally associating with another viral protein P22. Since LDs and the ER have been linked to several biological functions in cells, our study therefore proposes that the norovirus NTPase may utilize LDs or the ER as replication platforms to benefit viral replication and pathogenesis.

## INTRODUCTION

Noroviruses are nonenveloped, positive-sense, single-stranded RNA viruses belonging to the *Norovirus* genus of the *Caliciviridae* family (1). Noroviruses can be genetically subdivided into 10 genogroups (GI to GX); however, only GI, GII, and GIV infect humans and cause acute gastroenteritis (1, 2). Human noroviruses (HuNVs), especially the GII genotype 4 (GII.4) variants, are now recognized as the major cause of nonbacterial gastroenteritis worldwide (3–6). It is estimated that HuNVs cause around 699 million illnesses and 219,000 deaths across all ages annually (7). Despite the importance of HuNV in global gastroenteritis outbreaks, there is currently no licensed vaccine and specific antiviral treatment. Moreover, detailed study of viral replication and pathogenesis has also been hindered due to the absence of an efficient cell culture system or small animal model for HuNV propagation.

The complete RNA genome of HuNV is approximately 7.6 kb, which has a 3′ poly(A) tail and is covalently bound by a viral protein named “viral protein genome-linked (VPg)” at the 5′ end (8, 9). The viral genomic RNA contains three open reading frames (ORFs), namely, ORF1, ORF2, and ORF3 (8, 9). The viral ORF1 is translated as a large nonstructural polyprotein that can be further processed into six functional proteins. These nonstructural proteins include N-terminal nonstructural protein (Nterm or NS1-2), nucleoside-triphosphatase (NTPase or NS3), P22 (or NS4), VPg (or NS5), protease (Pro or NS6), and RNA-dependent RNA polymerase (RdRp or NS7). The viral ORF2 and ORF3 are responsible for the synthesis of the major capsid protein (VP1) and the minor capsid protein (VP2), respectively. Upon infection with HuNV, the incoming viral “genomic” RNA is thought to encode only the ORF1 polyprotein, which is then cleaved into six mature nonstructural proteins by Pro. These mature nonstructural proteins may directly or indirectly participate in the formation of the replication complex where RdRp serves as the key enzyme to catalyze the synthesis of the negative-sense RNA intermediate and later the “genomic” and “subgenomic” RNAs in host cells (8). During the progression of viral replication, a hallmark event that occurs in host cells is the remodeling of intracellular membranes induced by viral nonstructural proteins, which may facilitate the formation of viral replication compartments (10–12). At later stages of viral replication, the structural proteins VP1 and VP2 are translated from the “subgenomic” RNAs and are responsible for viral genome packaging. In comparison to extensive studies on viral structural proteins, there are very few studies on the roles of nonstructural proteins, particularly Nterm, NTPase, and P22, in viral replication and pathogenesis.

The HuNV-encoded NTPase typically contains 363 to 366 amino acid (aa) residues, which shares sequence similarity with superfamily 3 (SF3) viral helicases, such as poliovirus 2C protein, simian virus 40 (SV40) large T antigen, and human papillomavirus (HPV) E1 protein (13, 14). Previously, we have shown that the NTPase protein derived from a GII.4 strain (GII-NTPase) is able to interact with other viral proteins such as Nterm and P22 (15), suggesting that this protein may play a central role in the viral life cycle. Several biochemical studies have also demonstrated that norovirus NTPase possesses multiple enzymatic functions, including NTP-binding, NTP hydrolysis, ATP-dependent RNA helicase, and ATP-independent chaperoning activities (13, 14). Additionally, Cotton et al. (16) reported that the NTPase protein, derived from murine norovirus or a GI-norovirus strain (Norwalk virus), significantly induced the formation of vesicular structures in Vero cells (an African green monkey kidney cell line) and suggested that this viral protein could be a lipid- and microtubule-associated protein involved in viral RNA replication. Likewise, our previous studies also revealed that GII-NTPase could form both the vesicular and nonvesicular structures in A7 melanoma cells (15). Although the precise nature of vesicular structures formed by GII-NTPase remained unclear, our studies have shown that the nonvesicular fraction of GII-NTPase was colocalized with the endoplasmic reticulum (ER) or mitochondria (15). Deletion analysis further demonstrated that the N-terminal 179-aa region of GII-NTPase is the key domain for vesicular formation, ER localization, and interaction with Nterm and P22, whereas the C-terminal portion of GII-NTPase is responsible for localization to mitochondria (15). Intriguingly, we also found that the N-terminal portion, but not the C-terminal portion, of GII-NTPase possesses proapoptotic activity (15). Recently, in addition to the localization to the ER and mitochondria, Doerflinger et al. (12) have reported that norovirus NTPase could also associate with lipid droplets (LDs), an intracellular lipid storage organelle, in Huh7 cells. Since the norovirus NTPase appears to have multiple functions in viral replication and pathogenesis, a better understanding of its structure-function relationships may be important for providing opportunities to develop new antiviral strategies against the virus.

LDs are intracellular organelles that serve as reservoirs of neutral lipids, such as triacylglycerols (TAGs) and sterol esters (17, 18). They have a unique architecture that contains a neutral lipid core surrounded by a phospholipid monolayer and a group of specific surface proteins. LDs are derived from the ER (17, 18). With the synthesis and accumulation of TAGs and sterol esters in the ER membrane bilayer, nascent LDs are generated and subsequently bud from the ER membrane. The number, size, or distribution of LDs may vary greatly depending on cell types or cellular metabolic states (19). Due to the close association of LDs with other cellular organelles, such as the ER, mitochondria, nucleus, or peroxisomes, LDs may be substantially involved in a wide range of cellular processes, including lipid metabolism, energy supply, membrane biogenesis, proteostasis, and diverse signaling transduction pathways (18, 20). Moreover, a growing body of evidence also suggests that LDs play crucial roles in viral infection and pathogenesis (21, 22). Many experimental studies have demonstrated that several RNA viruses, including hepatitis C virus (23, 24), Dengue virus (25), enteroviruses (26–28), rotavirus (29), reovirus (30), and severe acute respiratory syndrome coronavirus 2 (SARS-CoV-2) (31), selectively modulate the biogenesis or functions of LDs to favor their own replication cycles. To date, although it has been reported that individual viral proteins of noroviruses could associate with LDs (12), it is not yet known whether there is a functional link between LDs and the replication cycle of noroviruses.

In this study, we aimed to characterize the sequence motifs of GII-NTPase required for vesicular formation, the induction of cellular apoptosis, and the physical and functional interaction with P22 in more detail. Here, we showed that the multivesicular structures bound by GII-NTPase are actually equivalent to cytosolic LDs, and two LD-targeting motifs, LTM-1 and LTM-2, have been identified in the N-terminal regions from aa 1 to 50 and from aa 51 to 90, respectively. The identified LTM-1 and LTM-2 motifs consist of a hydrophobic cluster and an amphipathic helix. Importantly, these two LD-targeting motifs could also confer the abilities to induce cellular apoptosis, to localize the fusion proteins to the ER, and to physically or functionally interact with P22. Collectively, our findings suggest that the norovirus NTPase may affect multiple cellular functions through binding to LDs or to the ER via its N-terminal amphipathic helix region.

## RESULTS

### The GII.4 norovirus NTPase predominantly localizes to lipid droplets and induces cell apoptosis through both caspase-8- and caspase-9-dependent pathways.

**FIG 1:**
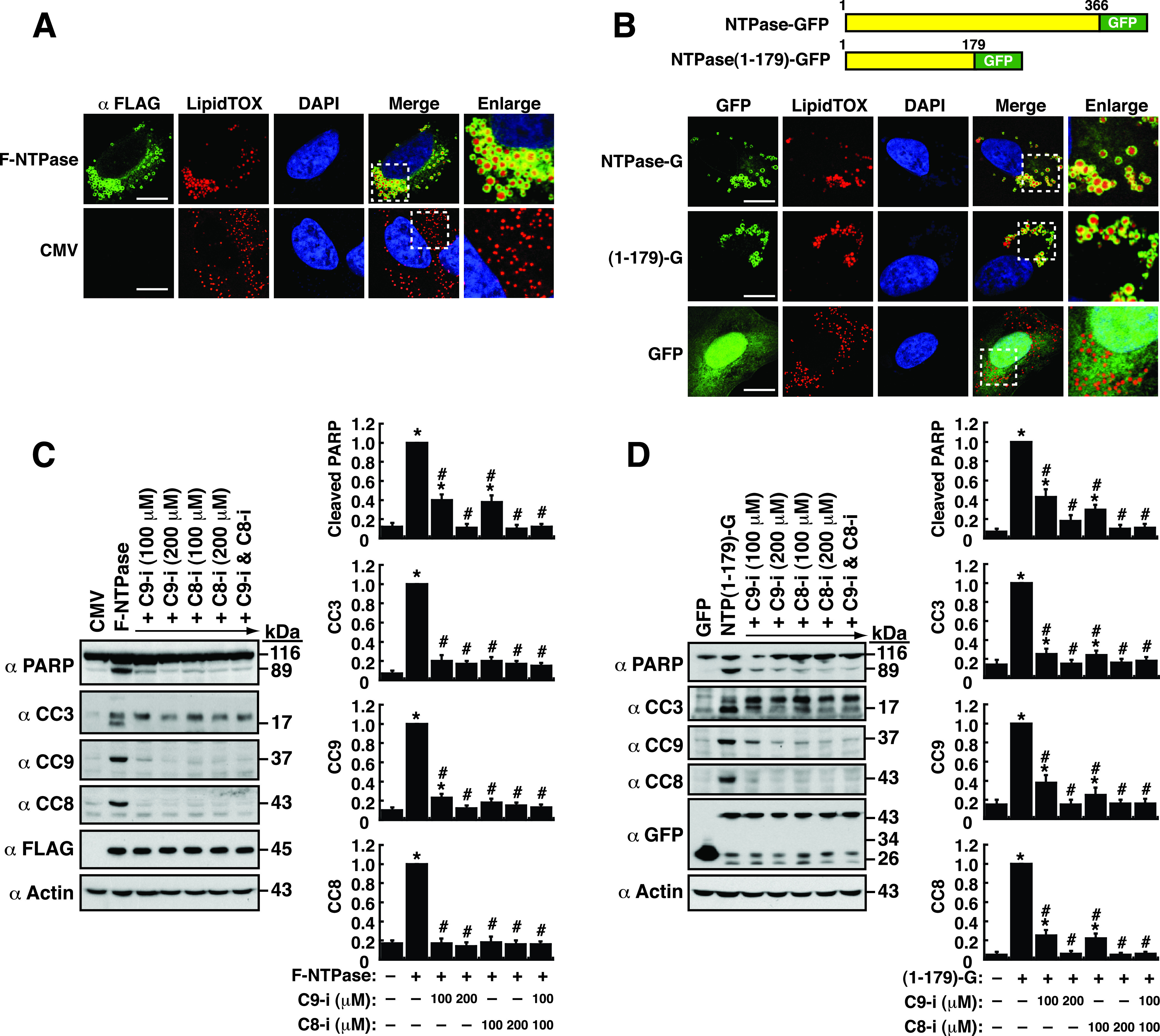
GII-NTPase predominantly localizes to the surface of lipid droplets (LDs) and induces cellular apoptosis through activation of both caspase-8- and caspase-9-dependent pathways. (A) Subcellular localization of F-NTPase and LDs in human melanoma A7 cells. Cells expressing F-NTPase were dually stained with an anti-FLAG antibody (green) and a fluorescent dye (LipidTOX Red) specific for LDs. The dashed boxes in the merged image were enlarged and are shown at the right; scale bars, 10 μm. (B) Association of the NTPase-GFP fusion constructs with LDs in A7 cells. Cells transfected with the expression plasmid encoding NTPase(1–366)-GFP, NTPase(1–179)-GFP, or GFP were stained with LipidTOX Red. The dashed boxes in the merged images were enlarged and are shown at the right; scale bars, 10 μm. (C and D) Effects of the caspase-8 or caspase-9 inhibitor on F-NTPase- or NTP(1–179)-GFP-mediated cellular apoptosis. 293T cells transfected with the indicated expression plasmids were untreated or treated with the caspase-9 inhibitor (C9-i; 100 or 200 μM), the capase-8 inhibitor (C8-i; 100 or 200 μM), or a combination of both inhibitors (100 μM each) for 24 h. The levels of cleaved PARP and caspases in treated cells were evaluated by Western blotting; CC3, cleaved caspase-3; CC8, cleaved caspase-8; CC9, cleaved caspase-9. The results, quantified in C and D, are expressed as mean ± standard error of the mean (SEM) from three independent experiments; *, *P* < 0.05 for results compared to those from the empty vector group (cytomegalovirus [CMV] or GFP); #, *P* < 0.05 for results compared to those from the untreated F-NTPase- or NTPase(1–179)-GFP-transfected group.

Previously, we have shown that the GII.4 norovirus-encoded NTPase contains multiple functional domains or motifs (15). Although the viral NTPase could potentially localize to the ER or mitochondria in transfected cells, the most prominent microscopic feature of this protein in cells is the formation of grape-like vesicles (Fig. 1A). These NTPase-associated vesicles are apparently not related to Golgi-derived secretory vesicles or endocytic vesicles (15, 16). When human melanoma A7 cells expressing a FLAG-tagged NTPase (F-NTPase) were stained with LipidTOX Red to reveal lipid droplets (LDs), we found that all LDs were perfectly wrapped by F-NTPase (Fig. 1A). In addition to F-NTPase, a full-length NTPase fused to green fluorescent protein (GFP), named NTPase-GFP, was also predominantly localized to the LD surface in A7 cells as detected by confocal microscopy (Fig. 1B). Since the N-terminal 179-aa region of NTPase was previously identified as the major region required for “vesicle formation,” the GFP fusion construct encompassing this region, NTPase(1–179)-GFP, was also tested for its association with LDs. Like NTPase-GFP, we observed that NTPase(1–179)-GFP, but not the control GFP, strikingly localized to LD surfaces in transfected cells (Fig. 1B). These results indicated that the N-terminal region of NTPase was sufficient to bind LD surfaces and form vesicle clusters.

Besides the ability to localize to LD surfaces, both the full-length NTPase and the NTPase(1–179) domain were known to promote cellular apoptosis in transfected cells (15). As shown in Fig. 1C and D, we confirmed that transfection of the plasmid expressing F-NTPase or NTPase(1–179)-GFP into 293T cells significantly induced the cleavage of poly(ADP-ribose) polymerase (PARP) and caspase-3, two known markers of apoptosis. Furthermore, apoptosis induced by NTPase(1–179)-GFP could also be verified by flow cytometry using Annexin-V and 7-aminoactinomycin D (7-AAD) staining (Fig. S1 in the supplemental material). During the course of the study, we additionally found that two apoptotic initiator caspases, caspase-8 and caspase-9, also underwent proteolytic cleavage in F-NTPase- or NTPase(1–179)-GFP-transfected cells (Fig. 1C and D). To determine the potential apoptotic pathway, namely, “intrinsic” or “extrinsic” pathway (32), mediated by NTPase or NTPase(1–179)-GFP, the transfected cells were treated with a caspase-9 inhibitor (Z-LEHD-FMK; 100 or 200 μM), a caspase-8 inhibitor (Z-IETD-FMK; 100 or 200 μM), or a combination of both inhibitors (100 μM each) (Fig. 1C and D). Western blotting showed that inhibition of either caspase-9 or caspase-8 activity in F-NTPase- or NTPase(1–179)-GFP-transfected cells markedly reduced their downstream apoptotic events, such as the cleavage of PARP and caspase-3 (Fig. 1C and D). Based on these results, F-NTPase- or NTPase(1–179)-GFP-induced apoptosis could be mediated through both the intrinsic (caspase-9-dependent) and extrinsic (caspase-8-dependent) signaling pathways.

### Deletion analysis of LD localization and apoptotic function of NTPase.

**FIG 2:**
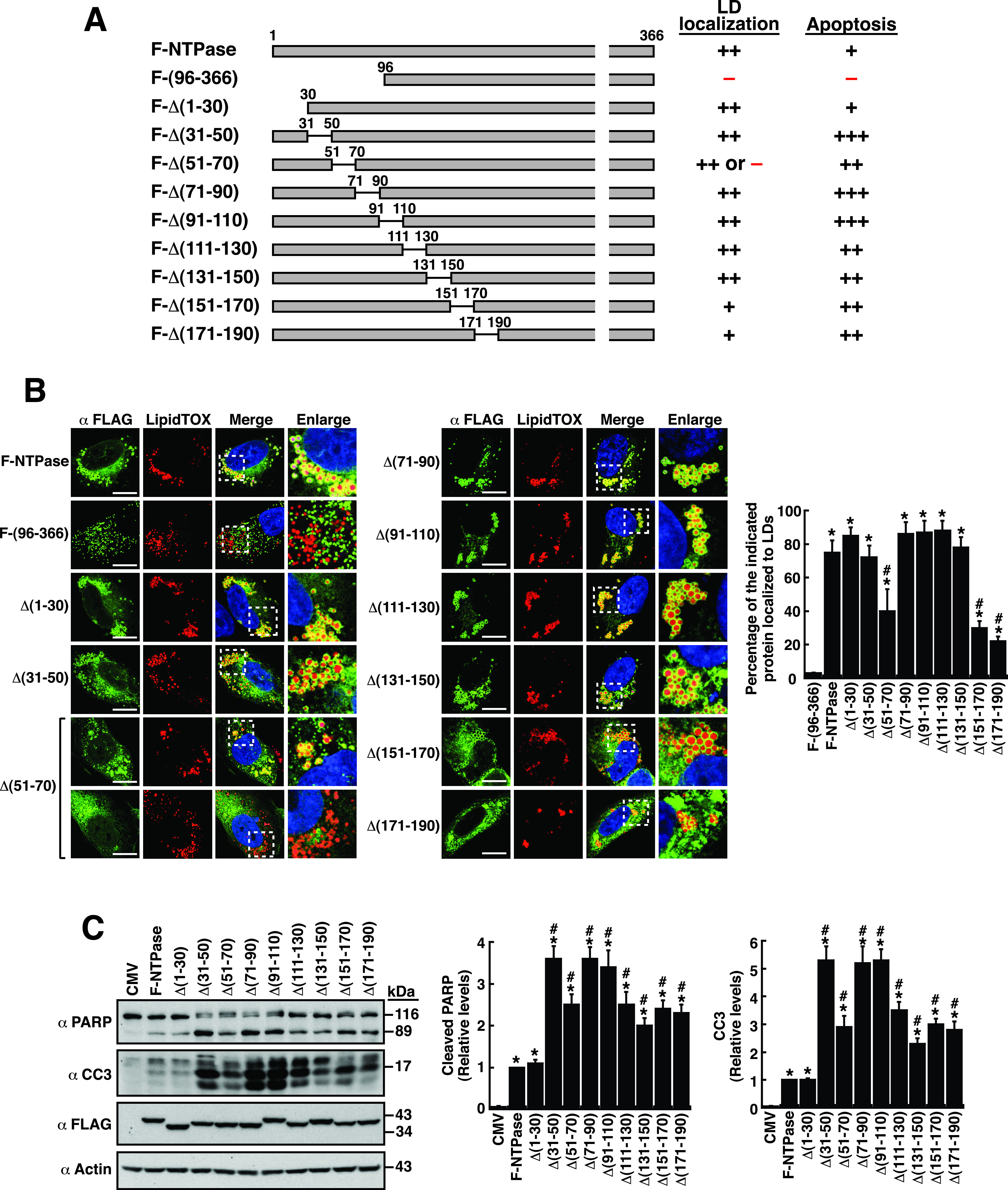
Deletion analysis of the LD localization and the apoptotic functions of F-NTPase. (A) Schematic diagram of F-NTPase mutants with a 30-aa or 20-aa deletion at the N-terminal region. The ability and degree of the F-NTPase deletion mutants to localize to LDs and to activate cellular apoptosis are summarized. (B) Confocal microscopic images showing the localization of F-NTPase deletion constructs and LipidTOX signals in A7 cells. The dashed boxes in the merged images were enlarged and are shown at the right; scale bars, 10 μm. Data are expressed as mean ± SEM (*n *= 15 cells obtained from 2 to 3 independent experiments); *, *P* < 0.05 for results compared to those from the F-(96–366)-transfected group; #, *P* < 0.05 for results compared to those from the F-NTPase-transfected group. (C) Western blotting analysis of the cleavage of PARP and caspase-3 induced by F-NTPase or its deletion mutants in 293T cells; CC3, cleaved caspase-3. As noted, the mutant proteins, including Δ(91–110) and Δ(131–150), migrated slower than the other deletion constructs with a similar size in SDS-PAGE gels. Data are expressed as mean ± SEM (*n *= 3); *, *P* < 0.05 for results compared to those from the empty vector group (CMV); #, *P* < 0.05 for results compared to those from the F-NTPase-transfected group.

To determine the region(s) of NTPase responsible for LD localization and for cellular apoptosis, we initially tested a set of F-NTPase deletion mutants that contain 30-aa or 20-aa deletions within the N-terminal domain. (Fig. 2A). Confocal microscopic analysis revealed that the wild-type F-NTPase predominantly localized to LD surfaces (“the LD-associated fraction”), with only a small pool of the protein in the cytosol (“the non-LD fraction”) (Fig. 2B). The mutant protein NTPase(96–366) that lacks the N-terminal 95-aa residues served as a negative control. Previously, we have shown that NTPase(96–366) expressed in cells was specifically colocalized with mitochondria (15). In the experiments, we found that the N-terminal 30-aa deletion, Δ(1–30), or several internal deletions, including Δ(31–50), Δ(71–90), Δ(91–110), Δ(111–130), and Δ(131–150), in F-NTPase did not cause appreciable changes in their LD-targeting ability (Fig. 2B). However, three internal deletions, including Δ(51–70), Δ(151–170), and Δ(171–190), in F-NTPase led to a reduction in the localization to LDs along with elevated levels of mutant proteins in non-LD areas (Fig. 2B). Peculiarly, the Δ(51–70) deletion mutant displayed normal LD localization in one half of transfected cells and little LD localization in the other half of transfected cells (Fig. 2B). The differential LD localization of the Δ(51–70) deletion mutant in transfected cells might be attributed to different metabolic states occurring in cells. Despite such changes, these internal deletions, including Δ(51–70), Δ(151–170), or Δ(171–190), did not completely lose their ability to localize to LD surfaces (Fig. 2B).

To examine the apoptotic potential of these F-NTPase deletion mutants, Western blotting was performed to assess the cleavage of PARP and caspase-3 in transfected cells. Compared to the wild-type F-NTPase, nearly all of the deletion mutants, except Δ(1–30) and NTPase(96–366), conversely displayed increased apoptotic activity (Fig. 2C). For the mutant protein Δ(1–30), apoptotic activity was similar to that of wild type (Fig. 2C). In contrast, the cleavage of PARP and caspase-3 was not detected in cells expressing the negative-control NTPase(96–366) (Fig. 2A and see below). In the testing, we additionally observed that two deletion mutants, including Δ(91–110) and Δ(131–150), in SDS-PAGE gels migrated slower than the other mutants with a similar size (Fig. 2C). The aberrant migration of these deletion mutants in SDS-PAGE gels might be related to changes in their local hydrophobicity or protein shape.

**FIG 3:**
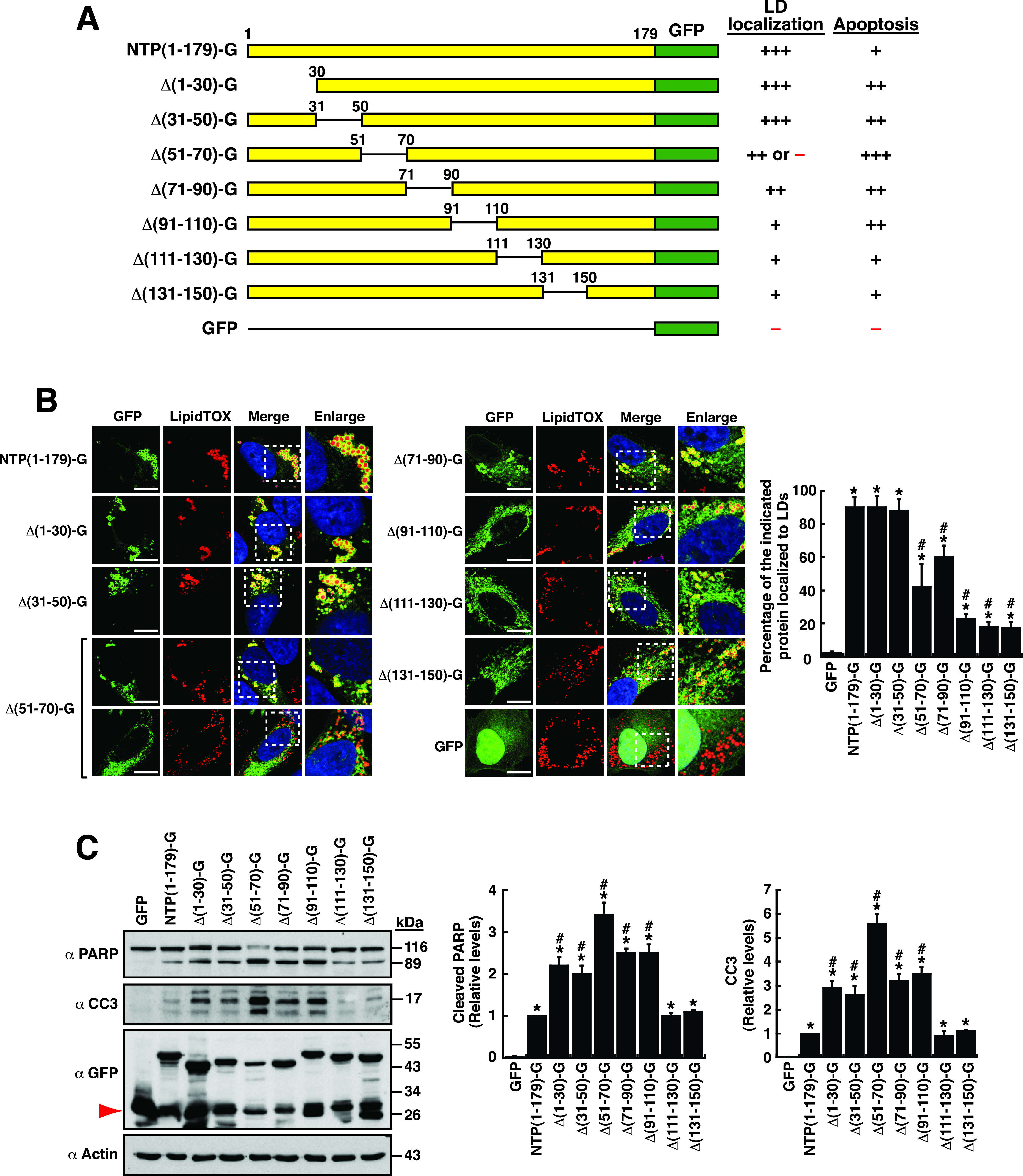
Deletion analysis of the LD localization and the apoptotic function of NTPase(1–179)-GFP. (A) Schematic diagram of NTPase(1–179)-GFP deletion mutants with a 30-aa or 20-aa deletion. The ability and degree of these NTPase(1–179)-GFP deletion mutants to localize to LDs and to induce cellular apoptosis are summarized. (B) Confocal microscopic images showing the localization of the indicated NTPase(1–179)-GFP deletion mutants and LipidTOX signals in A7 cells. The dashed boxes in the merged images were enlarged and are shown at the right; scale bars, 10 μm. Data are plotted as mean ± SEM (*n *= 15 cells obtained from 2 to 3 independent experiments); *, *P* < 0.05 for results compared to those from the empty vector group (GFP); #, *P* < 0.05 for results compared to those from the NTPase(1–179)-GFP-transfected group. (C) Western blotting analysis of the cleavage of PARP and caspase-3 induced by NTPase(1–179)-GFP and its deletion mutants in 293T cells; CC3, cleaved caspase-3. As noted, the mutant proteins, including Δ(91–110), Δ(111–130), and Δ(131–150), migrated slower than the other deletion constructs with a similar size in SDS-PAGE gels. The red arrowhead indicates a set of low-molecular-mass bands corresponding to the size of GFP concurrently detected in experimental groups. Quantitative data from Western blots are plotted as mean ± SEM (*n *= 3); *, *P* < 0.05 for results compared to those from the empty vector group (GFP); #, *P* < 0.05 for results compared to those from the NTPase(1–179)-GFP-transfected group.

Since we could not clearly define the LD-targeting or apoptosis-inducing regions in F-NTPase using the 30-aa or 20-aa deletion mutants, it was possible that the C-terminal domain of F-NTPase might carry the LD-targeting or apoptosis-inducing ability once specific N-terminal motifs were deleted. To test the possibility, similar 30-aa or 20-aa deletions were constructed in NTPase(1–179)-GFP (Fig. 3A), and their subcellular localization and their ability to induce apoptosis were evaluated by confocal fluorescence microscopy and Western blotting, respectively. We found that all of the NTPase(1–179)-GFP deletion mutants could still localize to LD surfaces, although some deletion mutants, including Δ(51–70)-GFP, Δ(71–90)-GFP, Δ(91–110)-GFP, Δ(111–130)-GFP, and Δ(131–150)-GFP, exhibited a lower extent of LD localization (Fig. 3B). We herein also noticed that the Δ(51–70)-GFP mutant could display two strikingly different localizations in transfected cells, where half of the transfected cells showed normal LD localization of this protein, while the other half showed no specific LD localization of the protein (Fig. 3B). It is likely that the localization of this Δ(51–70)-GFP construct to LD surfaces might be largely dependent on particular metabolic states in transfected cells. Moreover, similar to F-NTPase deletion mutants, nearly all of the NTPase(1–179)-GFP deletion mutants had enhanced proapoptotic activity (Fig. 3C). Based on these results, we postulate that the NTPase(1–179) region probably contains more than one LD-targeting or apoptosis-inducing motif.

Of note, as shown in the Western blotting analyses (Fig. 3C), several mutant proteins, such as Δ(91–110)-GFP, Δ(111–130)-GFP, and Δ(131–150)-GFP, migrated slower than the other deletion constructs with a similar size in SDS-PAGE gels. The aberrant migration of these deletion mutants in SDS-PAGE gels could be attributed to changes in their local hydrophobicity or protein shape. Additionally, we found that several low-molecular-mass products corresponding to the size of GFP, in addition to the expected GFP-tagged fusion proteins, could be concurrently detected in Western blots (Fig. 3C, red arrowhead). GFP cleavage from GFP fusion constructs probably occurred in cells under physiological conditions (e.g., apoptosis) or during the process of total protein extraction from cells (e.g., release of proteases/protease regulators, heat treatment, or sonication). We have attempted to find at which stage the GFP cleavage occurred. In this special case, when 293T or A7 cells were transfected with plasmids expressing NTPase(1–179)-GFP or its deletion constructs (with or without proapoptotic ability), the GFP cleavage event could be universally observed in Western blotting experiments (Fig. S2A and S2B). Since “free GFP” is a small protein and easily diffuses into the nucleus through the nuclear pores, we would expect to detect a prominent GFP fluorescent signal in the nucleus if the GFP cleavage occurs in growing cells that express NTPase(1–179)-GFP or its deletion constructs. However, the results from confocal microscopy experiments revealed that A7 cells transfected with the GFP-tagged fusion constructs, such as NTPase(1–179)-GFP or NTPase(31–179)-GFP, did not show any GFP signal in the nucleus (Fig. S2C). These results suggested that the GFP cleavage from these GFP-fused proteins probably did not occur in growing transfected cells, but it might occur at the process of total protein extraction from cells.

It should be noted that we have also cloned several other gene fragments (such as norovirus ORF2) into the same GFP-tagged vector (pEGFP-N2); however, none of them displayed the GFP cleavage according to Western blotting (data not shown). These results implicated that the fusion proteins, including NTPase(1–179)-GFP or its deletions, might have unique protein structures that make them susceptible to proteolytic cleavage at the sites between GFP and the NTPase(1–179) domain upon the preparation of protein extracts from cells.

### The NTPase(1–179) region contains two LD-targeting motifs, two apoptosis-inducing motifs, and multiple regulatory regions.

**FIG 4:**
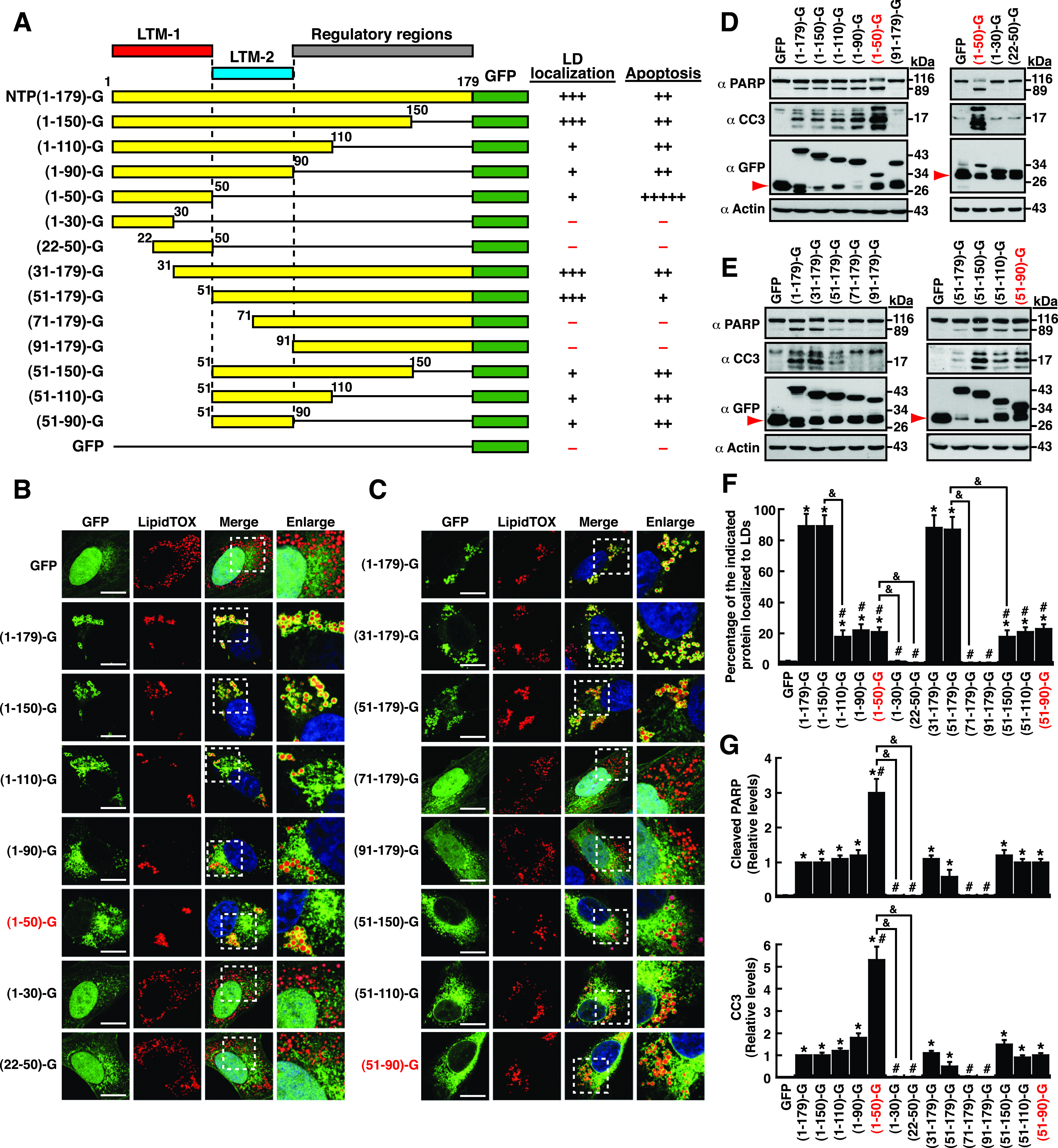
The NTPase(1–179) domain contains two LD-targeting or apoptosis-inducing motifs. (A) Diagram of a series of the N-terminal and C-terminal deletion mutants of NTPase(1–179)-GFP. The ability and degree of these NTPase(1–179)-GFP deletion constructs to localize to LDs and to induce cellular apoptosis are summarized; LTM-1, LD-targeting motif 1; LTM-2, LD-targeting motif 2. (B and C) Confocal microscopic images showing the localization of the indicated NTPase(1–179)-GFP deletion mutants and LipidTOX signals in A7 cells. The dashed boxes in the merged images were enlarged and are shown at the right; scale bars, 10 μm. (D and E) Western blotting analysis of the cleavage of PARP and caspase-3 induced by NTPase(1–179)-GFP or its deletion mutants in 293T cells. The red arrowheads indicate a set of low-molecular-mass bands corresponding to the size of GFP concurrently detected in experimental groups. (F) Quantitative analysis of LD localization of NTPase(1–179)-GFP and its deletion mutants. Data are presented as mean ± SEM (*n *= 15 cells obtained from 2 to 3 independent experiments); *, *P* < 0.05 for results compared to those from the empty vector group (GFP); #, *P* < 0.05 for results compared to those from the NTPase(1–179)-GFP-transfected group; &, *P* < 0.05, for results compared to those from the indicated group. (G) Quantitative analysis of the apoptotic potential of NTPase(1 to 179)-GFP and its deletion mutants in 293T cells. Data are presented as mean ± SEM (*n *= 3). *, *P* < 0.05 for results compared to those from the empty vector group (GFP); #, *P* < 0.05 for results compared to those from the NTPase(1–179)-GFP-transfected group; &, *P* < 0.05 for results compared to those from the indicated group.

In order to map a minimal region required for LD localization and for cellular apoptosis, a progressive series of the N-terminal or C-terminal deletions were generated in NTPase(1–179)-GFP (Fig. 4A). After the C-terminal deletion analysis, we found that the GFP fusion construct containing the protein fragment from aa 1 to 50, NTPase(1–50)-GFP, still retained the ability to localize to LDs (Fig. 4B), although the extent of NTPase(1–50)-GFP on LD surfaces (the LD-associated fraction) was lower than that of NTPase(1–179)-GFP (Fig. 4A, B, and F). In contrast, further deletions in NTPase(1–50)-GFP, such as NTPase(1–30)-GFP and NTPase(22–50)-GFP, completely eliminated the ability to localize to LD surfaces (Fig. 4A, B, and F). These results suggested that the NTPase(1–50) region was a functional LD-targeting motif. During the testing, we additionally noticed that the region from aa 110 to 150 of NTPase was required for the full activity of LD localization [Fig. 4A, B, and F, compare NTPase(1–110)-GFP and NTPase(1–150)-GFP], suggesting that this motif could be another LD-targeting motif or a regulatory motif essential for promoting LD localization.

On the other hand, when the N-terminal deletion mutants of NTP(1–179)-GFP were analyzed, we found that the mutants lacking the N-terminal 30- or 50-aa residues, NTPase(31–179)-GFP and NTPase(51–179)-GFP, still maintained a normal LD localization (Fig. 4A, C, and F). These results supported the notion that there was more than one LD-targeting motif in the NTPase(1–179) region. Further N-terminal deletion to aa 70 or aa 90 in NTPase(1–179)-GFP completely abolished the ability to localize to LD surfaces (Fig. 4A, C, and F). To map the second LD-targeting motif, a set of C-terminal deletions in NTPase(51–179)-GFP was generated, including NTPase(51–150)-GFP, NTPase(51–110)-GFP, and NTPase(51–90)-GFP (Fig. 4A). Fluorescence microscopic analysis revealed that the second LD-targeting motif was located to the region from aa 51 to 90 of NTPase [Fig. 4C, NTPase(51–90)-GFP]. Of note, we found that the C-terminal region from aa 150 to 179 might be involved in regulation of the activity of the second LD-targeting motif [Fig. 4A, C, and F, compare NTPase(51–179)-GFP and NTPase(51–150)-GFP]. According to these results, we concluded that the NTPase(1–179) region contains two LD-targeting motifs (designated LTM-1 and LTM-2), which are localized from aa 1 to 50 and from aa 51 to 90, respectively (Fig. 4A). Furthermore, although the C-terminal portion of NTPase(1–179) from aa 91 to 179 did not directly associate with LDs, this region might have roles in regulating LD-targeting activity.

To map the apoptosis-inducing region in NTPase(1–179), all the C-terminal and N-terminal deletion mutants (Fig. 4A) were analyzed for their abilities to induce the cleavage of PARP and caspase-3 in transfected cells. Interestingly, we found that the mutant proteins that failed to localize to LD surfaces, such as NTPase(1–30)-GFP, NTPase(22–50)-GFP, NTPase(71–179)-GFP, and NTPase(91–179)-GFP, could not induce the cleavage of PARP and caspase-3 (Fig. 4A to G). Herein, we identified two apoptosis-inducing motifs in NTPase(1–179)-GFP, which overlapped with the LTM-1 and LTM-2 motifs, respectively (Fig. 4A to G). Although the identified apoptosis-inducing motifs and the LD-targeting motifs were localized at the same places, we could not find a positive correlation between the extent of LD localization and the degree of cellular apoptosis for these NTPase(1–179)-GFP deletion mutants (Fig. 4A, F, and G). For example, NTPase(1–50)-GFP showed a lesser extent of LD localization than NTPase(1–179)-GFP; however, it had a much stronger apoptosis-inducing ability than NTPase(1–179)-GFP (Fig. 4A, F, and G). Additionally, LD localization of NTPase(51–90)-GFP was apparently lower than that of NTPase(1–179)-GFP, but NTPase(51–90)-GFP exhibited similar apoptosis-inducing ability to NTPase(1–179)-GFP (Fig. 4A, F, and G).

**FIG 5:**
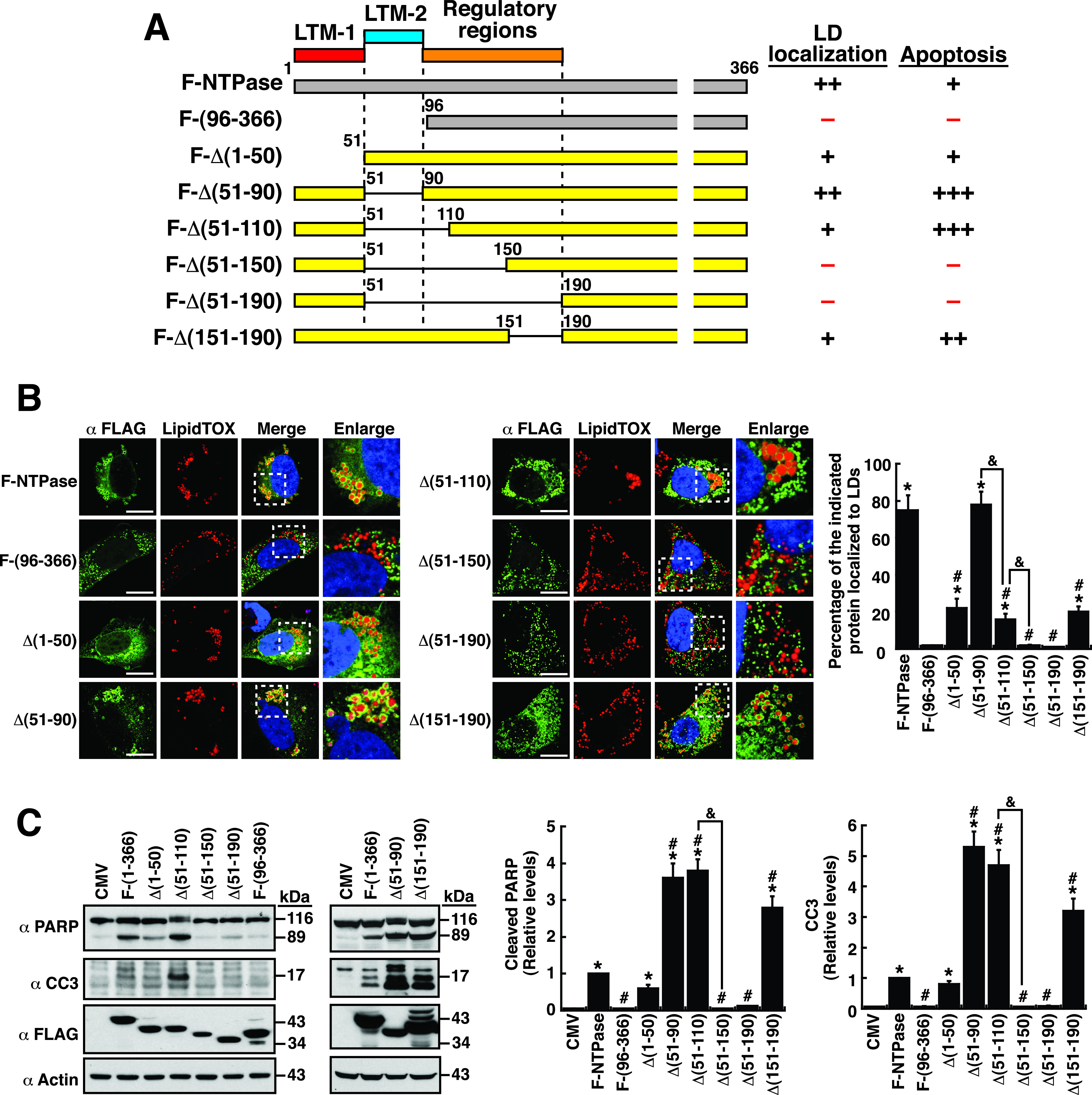
LTM-1, LTM-2, and multiple regulatory regions are involved in modulation of the LD-targeting and apoptosis-inducing functions of NTPase. (A) Diagram of F-NTPase deletion mutants. The ability and degree of F-NTPase deletion mutants binding to LDs and inducing cellular apoptosis are summarized; LTM-1, LD-targeting motif 1; LTM-2, LD-targeting motif 2. (B) Confocal microscopic images showing the localization of the indicated F-NTPase deletion constructs and LipidTOX signals in A7 cells. The dashed boxes in the merged images were enlarged and are shown at the right; scale bars, 10 μm. The quantitative results are plotted as mean ± SEM (*n *= 15 cells obtained from 2 to 3 independent experiments); *, *P* < 0.05 for results compared to those from the F-NTPase(96–366)-transfected group; #, *P* < 0.05 for results compared to those from the F-NTPase-transfected group; &, *P* < 0.05 for results compared to those from the indicated group. (C) Western blotting analysis of the cleavage of PARP and caspase-3 in 293T cells that were transfected with the indicated deletion constructs. The quantitative results are plotted as mean ± SEM (*n *= 3). *, *P* < 0.05 for results compared to those from the empty vector group (CMV); #, *P* < 0.05 for results compared to those from the F-NTPase-transfected group; &, *P* < 0.05 for results compared to those from the indicated group.

To further confirm the roles of LTM-1, LTM-2, and the regulatory regions for LD localization and cellular apoptosis, various deletion mutants were also generated in F-NTPase (Fig. 5A). Specific removal of the LTM-1 or LTM-2 motif from F-NTPase still retained the ability to localize to LD surfaces (Fig. 5A and B). However, an entire deletion of both the LTM-1 and LTM-2 motifs in F-NTPase, such as F-NTPase(96–366), led to a complete loss of the LD-targeting ability (Fig. 5A and B). Although deletion of the LTM-2 motif alone in F-NTPase did not compromise the ability to localize to LDs, further deletions across the LTM-2 motif, such as F-Δ(51–110), F-Δ(51–150), and F-Δ(51–190), markedly reduced LD localization of these mutant proteins (Fig. 5A and B). In particular, despite having the LTM-1 motif, the F-Δ(51–150)- and F-Δ(51–190)-mutant proteins did not show the ability to localize to LDs (Fig. 5A and B). These results supported the notion that multiple regulatory regions are involved in modulation of the LD-targeting activity of NTPase. Moreover, specific deletion from aa 151 to 190 in F-NTPase apparently resulted in reduced LD-targeting ability [Fig. 5A and B, F-Δ(151–190)]. Thus, in addition to the LTM-1 and LTM-2 motifs, specific regulatory regions in NTPase may also be required for full LD-targeting function. These regulatory regions are probably located within the region from aa 91 to 190 of NTPase.

When the proapoptotic activity of the F-NTPase deletion mutants was examined, we found that mutant proteins lacking the LTM-1 or LTM-2 motif, namely, F-Δ(1–50) and F-Δ(51–90), still retained the ability to induce cellular apoptosis (Fig. 5A and C). Similarly, we did not find a positive correlation between the extent of LD localization and the degree of cellular apoptosis for these F-NTPase deletion mutants (Fig. 5A and C). For example, the mutants, including F-Δ(51–90), F-Δ(51–110), and F-Δ(151–190) had higher proapoptotic activity than the wild-type F-NTPase, but they did not show higher LD-targeting ability than the wild-type F-NTPase (Fig. 5A to C). Despite lack of a positive correlation, we noticed that F-NTPase-mutant proteins that failed to localize to LDs, such as F-Δ(51–150), F-Δ(51–190), and F-(96–366), could not induce cellular apoptosis (Fig. 5A and C). Overall, these results implied that the LD-targeting ability of NTPase could be an essential prerequisite for triggering cellular apoptosis.

### The LTM-1 and LTM-2 motifs of NTPase have the ability to direct fusion proteins to the ER.

**FIG 6:**
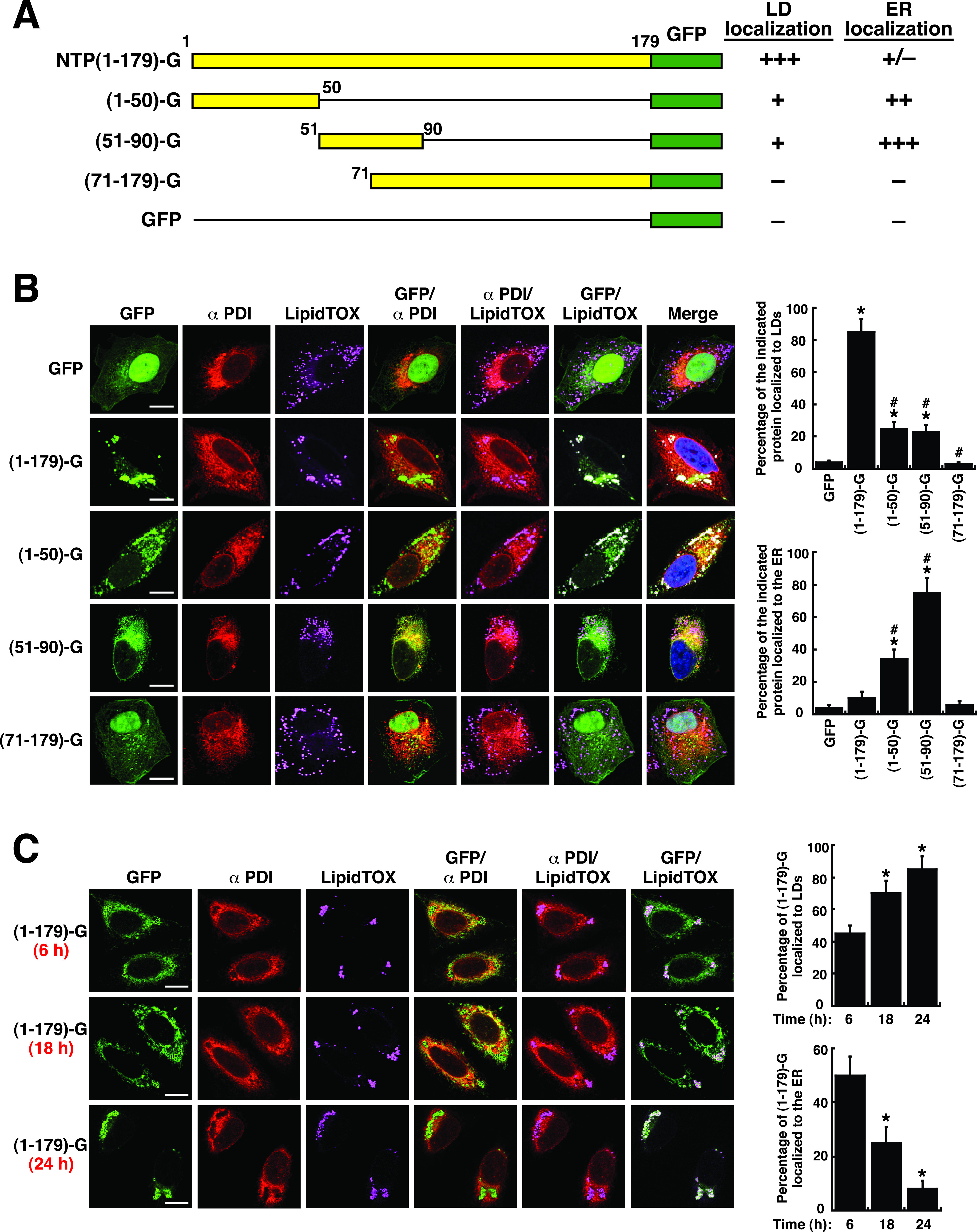
The LTM-1 and LTM-2 motifs of NTPase have the potential to direct fusion proteins to the ER. (A) Schematic diagram of various NTPase(1–179) deletion mutants. The ability and degree of NTPase(1–179)-GFP and its deletion mutants for LD localization and for ER localization are summarized. (B) Colocalization of NTPase(1–179) deletion constructs with LDs or with the ER in cells. A7 cells were transfected with the indicated expression plasmids for 24 h, and then the transfected cells were dually stained with LipidTOX and anti-PDI antibody; scale bars, 10 μm. The quantitative results are plotted as mean ± SEM (*n *= 15 cells obtained from two independent experiments); *, *P* < 0.05 for results compared to those from the empty vector group (GFP); #, *P* < 0.05 for results compared to those from the NTPase(1–179)-GFP-transfected group. (C) Confocal microscopic analysis of the localization of NTPase(1–179)-GFP in cells at different time points (6, 18, and 24 h) after transfection. A7 cells were transfected with the NTPase(1–179)-GFP expression plasmid for 6, 18, or 24 h, and then the subcellular localization of this protein to LDs or the ER in transfected cells was analyzed by confocal microscopy; scale bars, 10 μm. The quantitative results are plotted as mean ± SEM (*n *= 15 cells obtained from two independent experiments); *, *P* < 0.05 for results compared to those from the 6 h group.

As described above, although the GFP fusion proteins containing the LTM-1 or LTM2 motif, namely, NTPase(1–50)-GFP and NTPase(51–90)-GFP, could localize to LD surfaces, a considerable amount of these fusion proteins was also observed in non-LD areas (Fig. 4B and C). Due to the highly dynamic connection between LDs and the ER, we next investigated whether NTPase(1–50)-GFP or NTPase(51–90)-GFP was also localized to the ER. In the experiments, A7 cells were transfected with the GFP fusion constructs, including NTPase(1–179)-GFP, NTPase(1–50)-GFP, NTPase(51–90)-GFP, and NTPase(71–179)-GFP, for 24 h. The transfected cells were then dually stained with LipidTOX for LDs and an antibody specific for protein-disulfide isomerase (PDI), an ER marker (Fig. 6A and B). Under these conditions, NTPase(1–179)-GFP mainly localized to LD surfaces, and only a small fraction of the protein colocalized with PDI (Fig. 6A and B). The two negative controls, including GFP and NTPase(71–179)-GFP, colocalized to neither LDs nor the ER (Fig. 6A and B). Notably, a significant amount of NTPase(1–50)-GFP or NTPase(51–90)-GFP in A7 cells was found to colocalize with the signals of PDI (Fig. 6B, GFP/αPDI). These results indicate that the LTM-1 or LTM-2 motif has the potential to direct fusion proteins to the ER.

Since NTPase(1–179)-GFP was observed predominantly on LD surfaces, but not in the ER, in transfected cells, it raised the possibility that NTPase(1–179)-GFP might initially target to the ER (via the LTM-1 or LTM-2 motif) and then relocalize from the ER to LDs. To test this possibility, the subcellular localization of NTPase(1–179)-GFP was monitored in A7 cells at different time points after transfection. Confocal microscopic analysis revealed that at early time points (6 to 18 h) after transfection, a significant fraction of NTPase(1–179)-GFP was evidently visualized in the ER (Fig. 6C, GFP/αPDI). However, NTPase(1–179)-GFP tended to be localized to LD surfaces at later time points (18 to 24 h) after transfection (Fig. 6C). These results implied that the partition and dynamics of NTPase(1–179)-GFP to the ER or LDs might be profoundly modulated by the complicated associations among the LTM-1, LTM-2, and other regulatory motifs in the protein.

### The LTM region can interact both with itself and with the C-terminal portion of NTPase(1–179).

**FIG 7:**
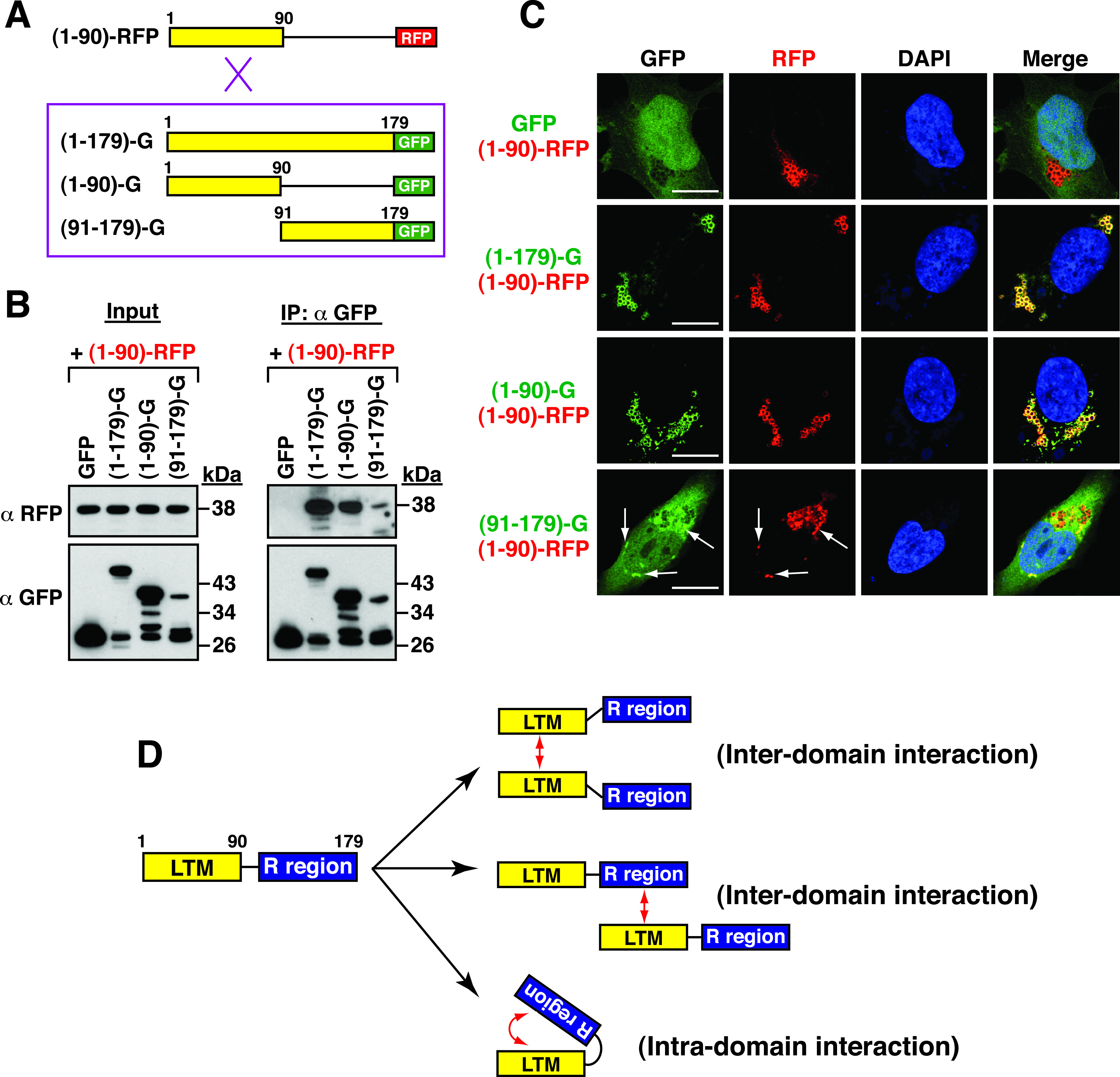
The N-terminal LTM region of NTPase(1–179) can interact with itself and with the C-terminal region of NTPase(1–179). (A) A schematic for analyzing the interaction of NTPase(1–90)-RFP with the NTPase(1–179)-GFP, NTPase(1–90)-GFP, or NTPase(91–179)-GFP construct. (B) Coimmunoprecipitation analysis of the interaction between NTPase(1–90)-RFP and different GFP fusion constructs in 293T cells. Protein lysates were prepared from transfected cells, and protein complexes were immunoprecipitated using anti-GFP antibody. The resultant immunoprecipitates were analyzed by Western blotting using anti-RFP or anti-GFP antibody. (C) Confocal microscopic analysis of the interaction between NTPase(1–90)-RFP and different GFP fusion constructs in A7 cells. Arrows indicate colocalization of NTPase(91–179)-GFP with NTPase(1–90)-RFP; scale bars, 10 μm. (D) Proposed model for the self-assembly of the LTM region and the intermolecular or intramolecular interaction between the LTM region and the C-terminal region of NTPase(1–179).

Previously, we have shown that the norovirus NTPase or NTPase(1–179) could self-assemble into dimeric or oligomeric structures (15). This prompted us to determine whether the LTM region from aa 1 to 90 could interact with itself. Additionally, since the C-terminal portion of NTPase(1–179) from aa 91 to 179 might have important roles in the regulation of LD localization and cellular apoptosis, the interaction between the LTM region and the C-terminal portion of NTPase(1–179) was also examined (Fig. 7A). In this study, a coimmunoprecipitation assay was performed using protein lysates from 293T cells that were cotransfected with a plasmid expressing a red fluorescent protein (RFP) fusion protein containing NTPase(1–90) [NTPase(1–90)-RFP] and a plasmid expressing GFP [NTPase(1–179)-GFP, NTPase(1–90)-GFP, or NTPase(91–179)-GFP] (Fig. 7A and B). After immunoprecipitation using anti-GFP antibody, NTPase(1–90)-RFP could be easily coimmunoprecipitated with NTPase(1–179)-GFP or NTPase(1–90)-GFP (Fig. 7B). In particular, the NTPase(1–90)-RFP protein was also coimmunoprecipitated with NTPase(91–179)-GFP, albeit at a lower level than that described above (Fig. 7B). Confocal microscopy analysis also confirmed that NTPase(1–90)-RFP perfectly colocalized with NTPase(1–179)-GFP or NTPase(1–90)-GFP and partially colocalized with NTPase(91–179)-GFP but did not colocalize with GFP (Fig. 7C). These results suggested that the LTM region is a self-assembling motif and can interact with the regulatory region located from aa 91 to 179 (Fig. 7D). It is worth noting that the interaction between the LTM region and the C-terminal regulatory region of NTPase(1–179) might occur in either an intermolecular or intramolecular manner (Fig. 7D).

### Functional characterization of mutations in the LTM-1 motif.

**FIG 8:**
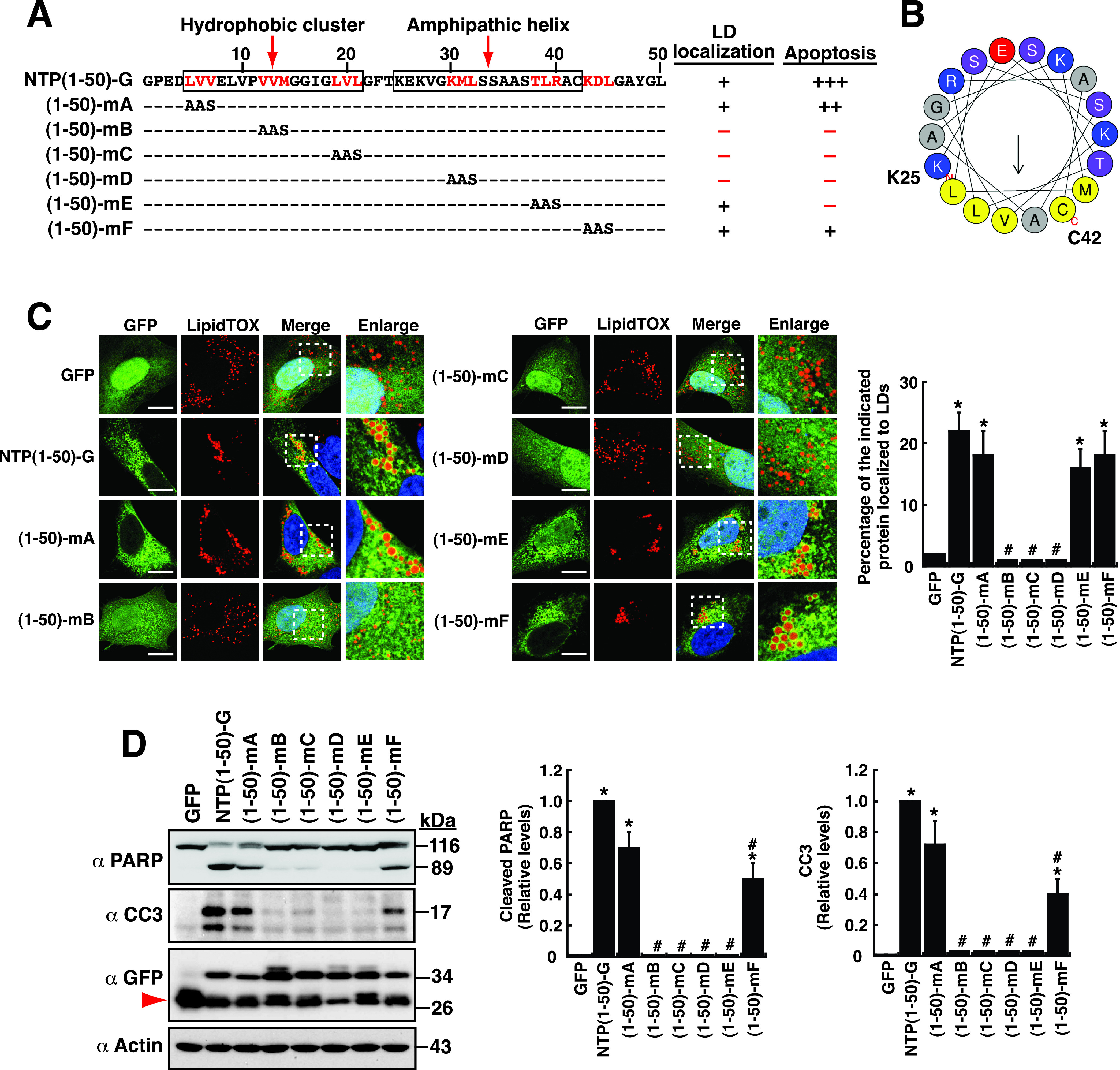
Functional characterization of point mutations in the LTM-1 motif. (A) Schematic diagram of wild-type and mutant forms of NTPase(1–50)-GFP. White boxes represent the positions of the hydrophobic cluster and the amphipathic helix predicted in the LTM-1 motif. The ability and degree of mutant constructs to localize to LD surfaces and to induce cellular apoptosis are summarized. (B) Helical wheeling drawing of the predicted amphipathic helix from aa 25 to 42. The amphipathic helix was drawn using HELIQUEST (https://heliquest.ipmc.cnrs.fr/cgi-bin/ComputParams.py). The hydrophobic face of the amphipathic helix contains amino acid residues M, C, A, V, L, and L. (C) Confocal microscopic images showing the localization of the indicated protein constructs and LipidTOX signals in A7 cells. The dashed boxes in the merged images were enlarged and are shown at the right; scale bars, 10 μm. The quantitative results are plotted as mean ± SEM (*n *= 15 cells obtained from three independent experiments); *, *P* < 0.05 for results compared to those from the empty vector group (GFP); #, *P* < 0.05 for results compared to those from the NTPase(1–50)-GFP-transfected group. (D) Western blotting analysis of the cleavage of PARP and caspase-3 induced by NTPase(1–50)-GFP and its deletion mutants in 293T cells. The quantitative results are plotted as mean ± SEM (*n *= 3); *, *P* < 0.05 for results compared to those from the empty vector group (GFP); #, *P* < 0.05 for results compared to those from the NTPase(1–50)-GFP-transfected group.

Sequence analysis and prediction of the LTM-1 motif of NTPase revealed that the N-terminal half from aa 5 to 21 contains a stretch of highly hydrophobic amino acids, whereas the C-terminal half from aa 25 to 42 forms an amphipathic helix (Fig. 8A and B). To further characterize the LTM-1 motif, point mutations were introduced into NTPase(1–50)-GFP (Fig. 8A). Since previous studies have reported that the large hydrophobic residues, including leucine (L), valine (V), methionine (M), isoleucine (I), and phenylalanine (F), in amphipathic helices are critical for lipid binding (33), the large hydrophobic residues in the LTM-1 motif were preferentially mutated. The resultant mutants include (1–50)-mA (LVV to AAS), (1–50)-mB (VVM to AAS), (1–50)-mC (LVL to AAS), (1–50)-mD (KML to AAS), (1–50)-mE (TLR to AAS), and (1–50)-mF (KDL to AAS). Confocal microscopic analysis revealed that some mutants, including (1–50)-mA, (1–50)-mE, and (1–50)-mF, still retained normal LD localization compared to the wild-type LTM-1 construct (Fig. 8A and C). However, some mutant proteins, including (1–50)-mB, (1–50)-mC, and (1–50)-mD, expressed in A7 cells were distributed predominantly in the nucleus and failed to localize to LD surfaces (Fig. 8A and C). We also found that the (1–50)-mB, (1–50)-mC, and (1–50)-mD mutant constructs entirely lost their ability to induce cellular apoptosis (Fig. 8A and D). Of note, despite a normal LD localization for the mutant (1–50)-mE, it could not induce cellular apoptosis (Fig. 8A and D). These results implied that the subcellular localization of NTPase to LDs was essential but probably not sufficient to induce cellular apoptosis.

### Functional characterization of mutations in the LTM-2 motif.

**FIG 9:**
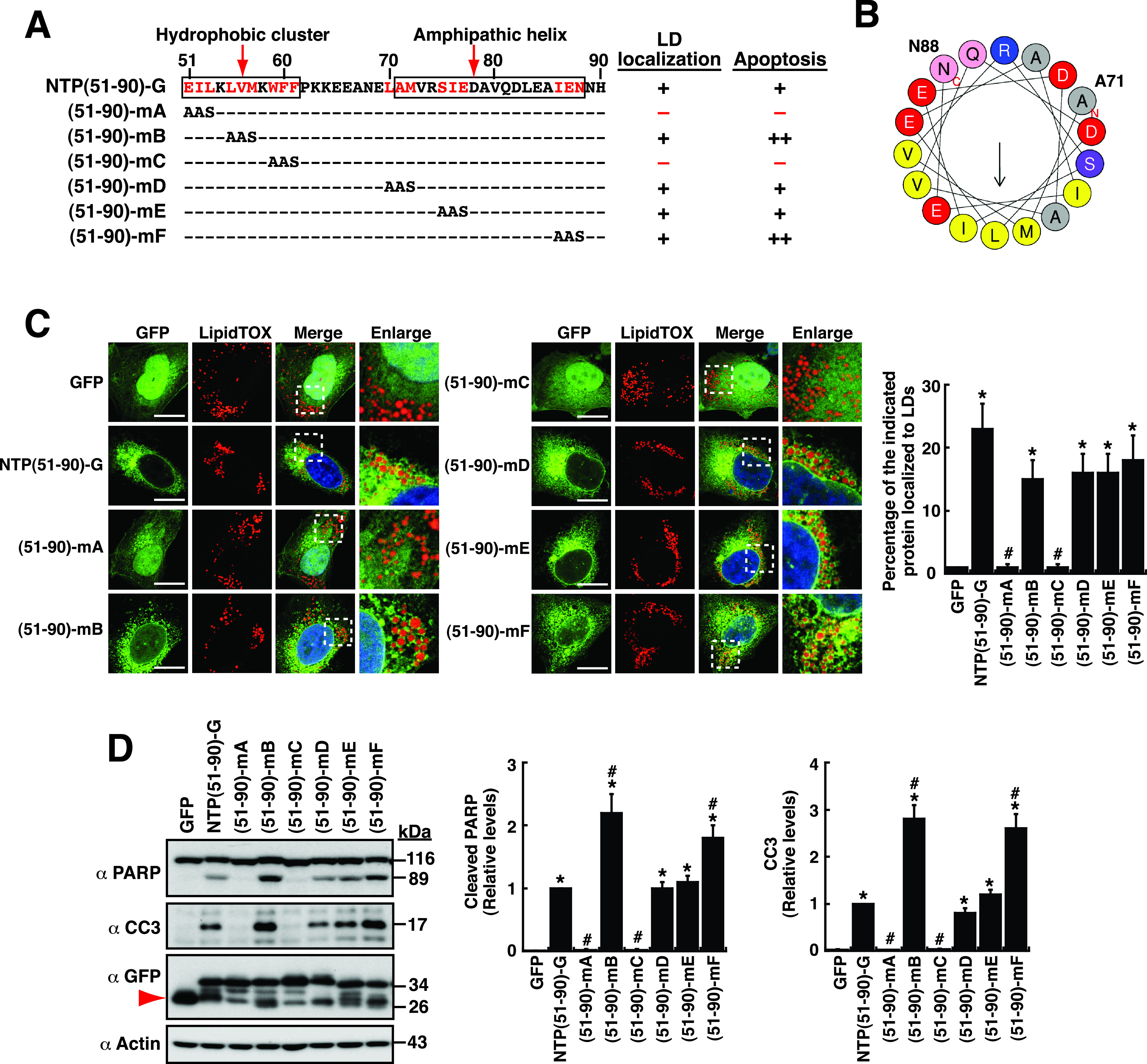
Functional characterization of point mutations in the LTM-2 motif. (A) Schematic diagram of wild-type and mutant forms of NTPase(51–90)-GFP. White boxes in the LTM-2 motif represent the positions of the hydrophobic cluster and the amphipathic helix predicted. The ability and degree of individual mutant constructs for LD localization and for cellular apoptosis are summarized. (B) Helical wheeling drawing of the predicted amphipathic helix from aa 71 to 88. The amphipathic helix was drawn using HELIQUEST (https://heliquest.ipmc.cnrs.fr/cgi-bin/ComputParams.py). The hydrophobic face of this amphipathic helix contains amino acid residues I, A, M, L, and I. (C) Confocal microscopic images showing the localization of the indicated GFP fusion constructs and LipidTOX signals in A7 cells. The dashed boxes in the merged images were enlarged and are shown at the right; scale bars, 10 μm. The quantitative results are plotted as mean ± SEM (*n *= 15 cells obtained from three independent experiments); *, *P* < 0.05 for results compared to those from the empty vector group (GFP); #, *P* < 0.05 for results compared to those from the NTPase(51–90)-GFP-transfected group. (D) Western blotting analysis of the cleavage of PARP and caspase-3 induced by NTPase(51–90)-GFP and its deletion mutants in 293T cells. The quantitative results are plotted as mean ± SEM (*n *= 3); *, *P* < 0.05 for results compared to those from the empty vector group (GFP); #, *P* < 0.05 for results compared to those from the NTPase(51–90)-GFP-transfected group.

Similarly, sequence analysis and prediction showed that the LTM-2 motif also contains a stretch of hydrophobic residues from aa 51 to 61 and an amphipathic helix from aa 71 to 88 (Fig. 9A and B). To further characterize the function of the LTM-2 motif in detail, point mutations were introduced in the NTPase(51–90)-GFP construct, specifically at the large hydrophobic amino acid residues of the LTM-2 motif (Fig. 9A). The resultant mutant proteins include (51–90)-mA (EIL to AAS), (51–90)-mB (LVM to AAS), (51–90)-mC (WFF to AAS), (51–90)-mD (LAM to AAS), (51–90)-mE (SIE to AAS), and (51–90)-mF (IEN to AAS) (Fig. 9A). Confocal microscopic analysis showed that the mutant proteins, including (51–90)-mB, (51–90)-mD, (51–90)-mE, and (51–90)-mF, still displayed normal LD localization compared to wild-type NTPase(51–90)-GFP (Fig. 9A and C). However, two mutant proteins, including (51–90)-mA and (51–90)-mC, failed to localize to LD surfaces (Fig. 9A and C). When the proapoptotic activity of these mutants was evaluated, we found that the mutants (51–90)-mA and (51–90)-mC also could not induce cellular apoptosis (Fig. 9A and D). Intriguingly, we noticed that the (51–90)-mB and (51–90)-mF mutants had enhanced proapoptotic activity compared to the wild-type NTPase(51–90)-GFP protein (Fig. 9A and D). These results suggested that both the hydrophobic motif and the amphipathic helix in the LTM-2 motif could play some roles for directing the fusion protein to the correct compartments and for proapoptotic activity.

### The LTM-1 and LTM-2 motifs are essential for the physical and functional interactions with P22.

**FIG 10:**
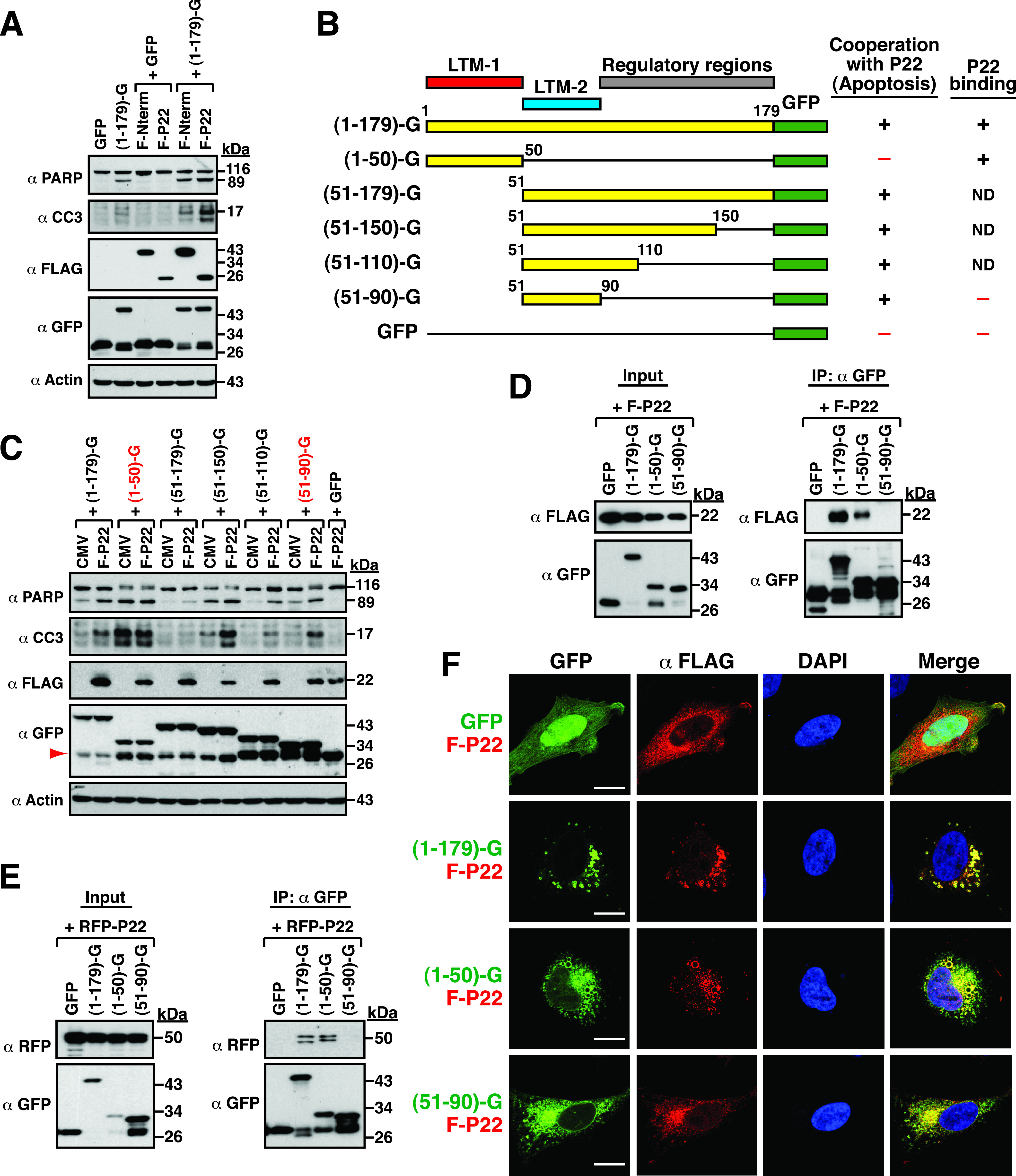
Functional and physical interactions between P22 and different protein fragments in NTPase(1–179). (A) Effects of F-Nterm or F-P22 on the proapoptotic activity of NTPase(1–179)-GFP. 293T cells were cotransfected with the indicated plasmids, and the expression of cleaved PARP and caspase-3 in transfected cells was evaluated by Western blotting. (B) Summary of the functional and physical interactions between different NTPase(1–179) fragments and P22; ND, not detected. (C) Effects of F-P22 on the proapoptotic activity of different NTPase(1–179)-GFP deletion constructs. After cotransfection of the indicated expression plasmids into 293T cells for 24 h, the expression of cleaved PARP and caspase-3 was examined by Western blotting. (D) Coimmunoprecipitation analysis of the interaction between different NTPase(1–179)-GFP deletion constructs and F-P22 in 293T cells. Cells were transfected with the F-P22 expression plasmid and the plasmid expressing GFP, NTPase(1–179)-GFP, NTPase(1–50)-GFP, or NTPase(51–90)-GFP for 24 h. The immunoprecipitation assay was performed using anti-GFP antibody, and the resulting immunoprecipitates were analyzed by Western blotting using anti-FLAG or anti-GFP antibody. (E) Coimmunoprecipitation analysis of the interaction between different NTPase(1–179)-GFP deletion constructs and RFP-P22 in 293T cells. All experimental conditions were the same as those described above except the RFP-P22 expression plasmid used here. (F) Confocal microscopic analysis of colocalization of F-P22 and various NTPase(1–179)-GFP fragments; scale bars, 10 μm.

Our previous studies have demonstrated that the viral Nterm and P22 proteins could individually interact with NTPase and augment the proapoptotic activity of NTPase (15). To determine whether Nterm or P22 enhanced the proapoptotic activity of NTPase(1–179)-GFP, 293T cells were cotransfected with the NTPase(1–179)-GFP expression plasmid and the plasmid expressing FLAG-tagged Nterm (F-Nterm) or FLAG-tagged P22 (F-P22). Western blotting analysis revealed that coexpression of F-Nterm or F-P22 with NTPase(1–179)-GFP significantly enhanced the proapoptotic activity of NTPase(1–179)-GFP (Fig. 10A). As noted, overexpression of F-Nterm or F-P22 alone in 293T cells did not cause cellular apoptosis (Fig. 10A). Due to the fact that F-P22 had a greater function to promote the proapoptotic activity of NTPase(1–179)-GFP than F-Nterm, we focused our study on the physical and functional interactions between F-P22 and the NTPase(1–179) domain. When various deletion constructs of NTPase(1–179)-GFP were cotransfected with F-P22 into 293T cells, we found that the proapoptotic activity of NTPase(1–50)-GFP could not be further enhanced by F-P22 (Fig. 10B and C). In contrast, the proapoptotic activity of all LTM-2-containing fusion constructs, including NTPase(51–179)-GFP, NTPase(51–150)-GFP, NTPase(51–110)-GFP, and NTPase(51–90)-GFP, was significantly augmented by F-P22 (Fig. 10B and C). These results indicated that P22 could functionally cooperate with the LTM-2 fusion construct, but not the LTM-1 fusion construct, to boost cellular apoptosis (Fig. 10B and C).

To investigate whether P22 physically interacted with specific protein fragments within NTPase(1–179), we initially performed coimmunoprecipitation experiments using the protein lysates of 293T cells that were cotransfected with the F-P22 expression plasmid and the plasmid expressing GFP, NTPase(1–179)-GFP, NTPase(1–50)-GFP, or NTPase(51–90)-GFP (Fig. 10D). After immunoprecipitation using anti-GFP antibody, we found that F-P22 was coimmunoprecipitated with NTPase(1–179)-GFP or NTPase(1–50)-GFP, but not NTPase(51–90)-GFP (Fig. 10D). These results were unexpected because F-P22 functionally cooperated with NTPase(51–90)-GFP, but not NTPase(1–50)-GFP, to promote cellular apoptosis. To further confirm the interaction between P22 and the LTM-1 motif, we additionally used the RFP-tagged P22 fusion construct, RFP-P22, in the coimmunoprecipitation assay. Consistently, the RFP-P22 fusion protein was also specifically coimmunoprecipitated by NTPase(1–50)-GFP but not by NTPase(51–90) (Fig. 10E). Furthermore, analysis of confocal microscopy images revealed that F-P22 noticeably colocalized with NTPase(1–179)-GFP and NTPase(1–50)-GFP (Fig. 10F). In particular, we noticed that the subcellular localization of F-P22 was remarkably changed in the presence of NTPase(1–179)-GFP or NTPase(1–50)-GFP, showing increased vesicle-like structures in cells (Fig. 10F). Although F-P22 and NTPase(51–90) seemed to be also localized to the same areas in transfected cells, no significant changes in the subcellular localization of F-P22 were observed in NTPase(51–90)-GFP-expressing cells compared to the control transfected cells (Fig. 10F). These results suggested that the functional cooperation between P22 and NTPase(51–90)-GFP might occur at the same subcellular compartment; however, this event did not need a direct protein-protein interaction.

## DISCUSSION

In this report, we have shown that the GII.4 norovirus-encoded NTPase is an LD-associated protein and can induce cellular apoptosis through both caspase-8- and caspase-9-dependent pathways. Furthermore, we have identified two LD-targeting motifs (LTM-1 and LTM-2) in NTPase, which are located at the N-terminal region from aa 1 to 50 and from aa 51 to 90, respectively. Both LTM motifs encompass a hydrophobic sequence cluster and an amphipathic helix. In addition to LD targeting, the identified LTM-1 and LTM-2 motifs in NTPase are also involved in ER localization, the induction of cellular apoptosis, protein dimerization or oligomerization, and interaction with another viral protein P22. Thus, our data suggest that the LTM-1 and LTM-2 motifs, along with other regulatory sequences, may play pivotal roles for many functions of NTPase in viral replication and pathogenesis.

### Subcellular localization of NTPase to LDs.

LDs are recognized as the central organelle responsible for lipid storage, lipid metabolism, lipid trafficking, energy supply, and various signaling transduction in cells (17, 18). Numerous RNA viruses have now been reported to use different strategies to modulate the biogenesis or functions of LDs to benefit their viral replication (23–31). Currently, it is completely unclear whether noroviruses manipulate LDs to favor the viral replication cycle. Here, we propose that the viral NTPase is an LD-binding protein that can further promote LD clustering in cells (Fig. 1A and B). Due to the fact that a hallmark event for HuNV replication is the remodeling of intracellular membranes, our findings therefore suggest that LDs may be part of membrane-associated viral replication compartments. Additionally, since LDs are involved in multiple cellular processes, the association between the viral NTPase and LDs may influence some specific LD-mediated functions. Indeed, our confocal microscopy analysis has revealed that in addition to LD clustering, NTPase or NTPase(1–179) could also substantially affect the number and size of LDs in transfected cells (Fig. S3 in the supplemental material). Specifically, larger sizes and lower numbers of LDs are often seen in NTPase- or NTPase(1–179)-transfected cells compared to in control vector-transfected cells (Fig. S3). Although the molecular mechanism of how NTPase regulates LD numbers and sizes remains unclear, we speculate that environmental conditions (e.g., deprivation or overload of nutrients or fatty acids) could also be other critical factors for the changes of LD sizes and numbers by NTPase. In future research, it will be important to understand whether NTPase affects LD biogenesis and functions under different conditions such as fatty acid overload or deprivation.

According to the known routes of proteins targeting to LDs, the LD-associated proteins can be generally grouped into two classes, namely, class I and class II (20, 34). Members of class I proteins are membrane-embedded proteins initially localized at the ER that relocalize from the ER to LD surfaces through membrane bridges. Typically, class I proteins have a dual localization in the ER and on LDs. On the other hand, class II proteins directly associate with LD surfaces from the cytosol after the protein is synthesized. Although several studies have attempted to find the conserved sequences of LD-targeting signals from cellular or viral proteins, most of the known LD-targeting motifs display highly variable amino acid sequences and sequence lengths. In general, these identified LD-targeting motifs contain either hydrophobic clusters (mainly found in class I proteins) or amphipathic helices (mainly found in class II proteins). Although the precise pathway for the localization of NTPase to LD surfaces is still unclear, our results suggest that NTPase may probably belong to a member of class I proteins. First, we noticed that the identified LD-targeting motifs (LTM-1 and LTM-2) have an intrinsic ability to bind to the ER (Fig. 6B). Second, when NTPase(1–179)-GFP was transiently expressed in cells, we found that a considerable amount of this protein resided in the ER at early time points after transfection (Fig. 6C).

Despite the critical role of the LTM-1 and LTM-2 motifs in directing NTPase to LDs or the ER, multiple auxiliary regulatory regions in NTPase may also be required for controlling the partitioning of this protein to LDs or to the ER. Our deletion analysis has revealed that several regulatory regions are located within the C-terminal portion of NTPase(1–179) from aa 90 to 179. Indeed, deletion of this regulatory region from aa 91 to 179 in NTPase(1–179)-GFP significantly reduced the extent of LD localization, along with increased amount of the mutant protein in non-LD areas (Fig. 4). Since the regulatory region from aa 91 to 179 could interact with the LTM region as detected by coimmunoprecipitation and confocal microscopy (Fig. 7), our results strongly suggest that the intramolecular interaction among the LTM-1, LTM-2, and regulatory motifs in NTPase may serve as an important strategy to control protein functions (Fig. 7D). During the course of the study, we additionally found that the LTM region can self-assemble into dimeric or oligomeric complexes, which may partly explain why NTPase or NTPase(1–179)-GFP promotes LD clustering. Although the significance of the LD or ER localization of NTPase in viral replication or pathogenesis is still far elusive, we think that the localization of NTPase to LDs or to the ER may have impacts on lipid metabolism and intracellular trafficking pathways in host cells.

### NTPase-associated cellular apoptosis.

Previously, several studies have documented that infection with caliciviruses, such as HuNV, murine norovirus (MNV), or feline calicivirus (FCV), substantially causes cell apoptosis (35–37). Although the exact mechanism of norovirus-induced apoptosis is not fully understood, activation of caspase-3, caspase-8, and caspase-9 could be found in MNV- or FCV-infected cells (36, 37). Importantly, Herod et al. (38) have also shown that ectopic expression of the MNV ORF1 polypeptide in HEK293 cells was able to sufficiently induce cellular apoptosis. In the present study, we report that the expression of HuNV NTPase alone in 293T cells is capable of activating cellular apoptosis through both caspase-8- and caspase-9-dependent pathways. To our knowledge, this is the first study to show the importance of nororviral NTPase in activating the caspase-8- and caspase-9-dependent apoptotic pathways in cells. However, due to the potential crosstalk between the caspase-8-mediated (extrinsic) and caspase-9-mediated (intrinsic) apoptotic pathways (39, 40), the detailed signaling cascade for apoptosis induced by NTPase still remains to be determined. Although it is unclear how NTPase activates the signaling cascade of apoptosis, we have identified two apoptosis-inducing motifs localized within the N-terminal region of NTPase.

Despite the overlap between the apoptosis-inducing motifs and LD-targeting motifs, we did not find a positive correlation between the extent of LD localization and the degree of cellular apoptosis. In particular, we showed that NTPase(1–50)-GFP, which had much better proapoptotic activity than NTPase(1–179)-GFP, did not exhibit a higher degree of LD localization than NTPase(1–179)-GFP in cells (Fig. 4). Due to the observation that some specific mutants of NTPase(1–179) could localize to both LDs and the ER, it raises the possibility that dynamic partitioning of mutant proteins to the ER or LDs may substantially contribute to cellular apoptosis. Accordingly, specific localization of proteins to LDs and to the ER may influence both lipid homeostasis and cellular trafficking pathways, respectively, ultimately leading to activation of both caspase-8-dependent (extrinsic) and caspase-9-dependent (intrinsic) apoptotic pathways. Of note, the NTPase or NTPase(1–179) deletion mutants, which were defective in LD localization, also could not localize to the ER and induce cellular apoptosis (Fig. 4 and 5). Consistently, point mutation analysis of the LTM-1 or LTM-2 motif also revealed that elimination of the LD-targeting ability in mutant constructs dramatically reduced their proapoptotic activity (Fig. 8 and 9). These findings strongly suggest that the LD-targeting and/or ER-targeting activity of NTPase could be an essential prerequisite for inducing cellular apoptosis.

### Physical and functional interactions between NTPase and P22.

In addition to self-assembly, the norovirus NTPase may interact with other viral or cellular proteins to enforce its diverse and complicated functions. We have previously reported that the viral P22 protein could interact with NTPase and enhance the proapoptotic activity of NTPase. Herein, we further characterized the functional and physical interactions between P22 and the NTPase(1–179) domain. Surprisingly, we found that the enhancement of the proapoptotic activity of NTPase(1–179) by P22 is independent of their physical interaction (Fig. 10B to F). Although the LTM-1 motif was identified as a P22-interacting motif in NTPase(1–179), P22 could not further boost the proapoptotic activity of the LTM-1 fusion protein (Fig. 10C). In contrast, despite no direct interaction between P22 and the LTM-2 motif, P22 substantially enhanced the proapoptotic activity of the LTM-2-containing fusion constructs (Fig. 10B to F). Of note, although the LTM-2 fusion protein could not interact with P22 as detected by coimmunoprecipitation assays, we did find that both the LTM-2 fusion protein and P22 frequently localized to the same areas (most likely at the ER) in cells. Although the underlying mechanism remains elusive, we here propose that the functional cooperation between P22 and the LTM-2 fusion protein may largely depend on their dynamic colocalization at specific intracellular organelles.

Despite the efforts in elucidating the possible functions of HuNV NTPase in cells, there are several critical limitations to this current study. First, here, we characterized only the potential functions of NTPase expressed individually but not in the context of viral replication. It is therefore clear that only some specific functions of the viral NTPase could be detected in the study. In the case of poliovirus, Egger et al. (41) have previously shown that even after a typical induction of membrane modification and vesicle budding by the transfected nonstructural viral proteins, these modified membranes could not be incorporated into the functional replication complexes built up by the subsequent poliovirus superinfection. This study implied that the overall functions of a given protein in viral replication or pathogenesis may rely on not only other viral proteins but also the replicating viral RNA in *cis* at the target cell (41). In the future, to further investigate the function of NTPase or the significance of specific motifs of NTPase in viral replication and pathogenesis, it is important to establish a reverse genetics system in the context of a full viral genome. Second, the cell types (including A7 and 293T cells) used in the study are not fully permissive to HuNV replication. Thus, to further validate the relationship between the viral NTPase and LDs, future work needs to be performed in naturally permissive cells. Previously, a nontransformed stem cell-derived human intestinal enteroid (HIE) culture was reported as an excellent model to support robust cultivation of multiple HuNV strains (42). In addition to HIEs, efficient propagation of HuNV has also been described recently in human induced pluripotent stem cell (iPSC)-derived intestinal epithelial cells (43) and in zebrafish (Danio rerio) larvae (44). These robust and physiologically relevant cultivation systems may aid in determining whether HuNV infection affects the LD size, number, and/or localization in infected cells and whether modulation of lipid metabolism in susceptible cells (enterocytes) influences HuNV infection.

In summary, we have identified two novel functional motifs, LTM-1 and LTM-2, in the N-terminal region of NTPase. Both motifs contain a hydrophobic cluster and an amphipathic helix. Besides serving as LD-targeting sequences, we here demonstrate that the LTM-1 and LTM-2 motifs are also required for ER localization, cellular apoptosis, molecular self-assembly, as well as the functional and physical interactions with the viral P22 protein. Our findings therefore highlight that NTPase may participate in multiple cellular processes via the N-terminal amphipathic helix motifs.

## MATERIALS AND METHODS

### Cell cultures, transfections, and chemical reagents.

Human melanoma cell line A7 (45) and human embryonic kidney cell line 293T (46) were cultured in high-glucose Dulbecco’s modified Eagle’s (DME) medium (11965084, Thermo Fisher Scientific) supplemented with 10% fetal bovine serum (10437028, Thermo Fisher Scientific). All transfection experiments were conducted using Lipofectamine 2000 transfection reagent (11668019, Thermo Fisher Scientific) according to the manufacturer’s instructions. Typically, A7 cells (1.5 × 10^5^ cells/well) and 293T cells (8 × 10^5^ cells/well) were seeded in 6-well culture plates a day before transfection and were transfected with 1 μg of plasmid DNA (mixed with 2.5 μl of Lipofectamine 2000) and 3 μg of plasmid DNA (mixed with 7.5 μl of Lipofectamine 2000), respectively. The concentrations of the caspase-9 inhibitor Z-LEHD-FMK (1149-5, BioVision) and the caspase-8 inhibitor Z-IETD-FMK (1148-5, BioVision) used in the study were 100 μM or 200 μM.

### Plasmid construction.

The expression plasmids, including pCMV-F-NTPase, pCMV-F-P22, and pCMV-F-Nterm, have been described previously (15). The plasmids pCMV-NTPase-GFP and pCMV-NTPase(1–179)-GFP were constructed by inserting the coding regions of NTPase and NTPase(1–179), respectively, into pEGFP-N2 (6081-1, Clontech). To obtain the series of the N- or C-terminal deletions of NTPase(1–179) in pCMV-NTPase(1–179)-GFP, the corresponding DNA fragments were amplified by PCR and then cloned into pEGFP-N2. The mutant constructs that contain internal deletions in pCMV-F-NTPase and pCMV-NTPase(1–179)-GFP were generated by using inverse PCR. Point mutations in pCMV-NTPase(1–50)-GFP or pCMV-NTPase(51–90)-GFP were created using a QuickChang site-directed mutagenesis kit (200524, Agilent Technologies). The plasmid that encodes NTPase(1–90)-RFP was constructed by inserting the RFP-coding fragment downstream of the NTPase(1–90)-coding sequences in pFLAG-CMV-2 (E7398, Sigma-Aldrich).

### Western blotting analysis.

All the steps of the Western blotting analysis have been described previously (47). Briefly, cells were lysed in either Laemmli sample buffer containing 62.5 mM Tris-HCl (pH 6.8), 2% SDS, 10% glycerol, and 5% 2-mercaptoethanol or in immunoprecipitation assay buffer containing 50 mM Tris-HCl (pH 7.6), 150 mM NaCl, 1 mM EDTA, 1% Triton X-100, and 1 mM phenylmethylsulfonyl fluoride. The protein lysates were then subjected to 8 to 12% SDS-PAGE followed by immunoblotting with specific antibodies. Antibodies specific to PARP (9532, Cell Signaling), cleaved caspase-3 (9664, Cell Signaling), cleaved caspase-9 (72375, Cell Signaling), cleaved caspase-8 (9496, Cell Signaling), FLAG (A8592, Sigma), GFP (G1544, Sigma), RFP (Sab2702214, Sigma), and actin (sc-47778, Santa Cruz) were purchased commercially. To quantify the relative levels of target proteins in Western blots, densitometry analyses of protein bands were performed using Quantity One software (Bio-Rad), and β-actin was used as a normalizing protein.

### Confocal immunofluorescence.

Immunofluorescence staining and confocal microscopic analysis were performed as described previously (15). Normally, A7 melanoma cells grown on coverslips were transiently transfected with the expression plasmids. Typically, after cultivation for 24 h, the transfected cells were fixed with 4% paraformaldehyde in phosphate-buffered saline (PBS) at room temperature for 10 min. For LD staining, cells were incubated with LipidTOX stain (H34476, Invitrogen) at 37°C for 15 to 20 min prior to cell fixation. The fixed cells were subsequently permeabilized with 0.1% Triton X-100 in PBS for 8 min at room temperature. Following incubation with blocking solution (CAS-Block, Invitrogen) for 30 min, cells were incubated with specific primary antibodies for 1 h. The primary antibodies against FLAG (F1804 or F7425, Sigma) and PDI (MA3-019, Thermo Fisher Scientific) were obtained commercially. After the slides were washed three times with PBS for 5 min each, cell samples were incubated with appropriate secondary antibodies. Staining with 4′-6-diamidino-2-phenylindole (DAPI) was conducted at room temperature for 15 min. The slides were mounted, and cells were analyzed using a confocal laser scanning microscope (Leica TCS-SP5II). The confocal images were acquired using LAS AF Lite software (Leica) under the same settings. To determine the degree of colocalization of target proteins with LDs or the ER, the Mander’s overlap coefficient was measured using the plugin Coloc2 (version 2.1.0) of the Image J software (version 1.52p; National Institutes of Health, Bethesda, MD). Prior to running Coloc2 in Image J, images with multiple color channels were split and then converted to 8-bit grayscale images. The Mander’s tM2 coefficients of channel 2 (GFP- or FLAG-tagged proteins) overlapping with channel 1 (LipidTOX or PDI) in images above autothreshold of channel 1 were calculated.

### Coimmunoprecipitation.

Coimmunoprecipitation assays were performed as described previously (48). Typically, 293T cells were cotransfected with plasmids expressing GFP fusion proteins and the plasmids expressing RFP-tagged or FLAG-tagged proteins. Cells were harvested 24 h after transfection and then lysed in the immunoprecipitation assay buffer (50 mM Tris-HCl pH 7.6, 150 mM NaCl, 1 mM EDTA, 1% Triton X-100, and 1 mM phenylmethylsulfonyl fluoride). Subsequently, protein lysates were subjected to immunoprecipitation using anti-GFP magnetic beads (gtma-20, ChromoTek). After immunoprecipitation, the resultant immunoprecipitates were examined by Western blotting using specific antibodies.

### Statistical analysis.

All data were presented as mean ± standard error of mean (SEM). The Mann-Whitney *U* test was used to evaluate differences between samples. All statistical analyses were performed using Statistical Package for the Social Sciences (SPSS) software (version 18.0; IBM Corporation, Armonk, NY). *P* values less than 0.05 were considered statistically significant.
