# Supplementary material for: Identification and Characterization of Human Norovirus NTPase Regions Required for Lipid Droplet Localization, Cellular Apoptosis, and Interaction with the Viral P22 Protein
Source: Microbiol Spectr. 2021 Aug 25;9(1):10.1128/spectrum.00422-21. doi: 10.1128/spectrum.00422-21 (PMC8552650; doi:10.1128/spectrum.00422-21)
Supplement: SUPPLEMENTAL FILE 1 — Supplemental material. Download SPECTRUM00422-21_Supp_1_seq14.pdf, PDF file, 1.4 MB [file spectrum00422-21_supp_1_seq14.pdf]

SUPPLEMENTAL INFORMATION

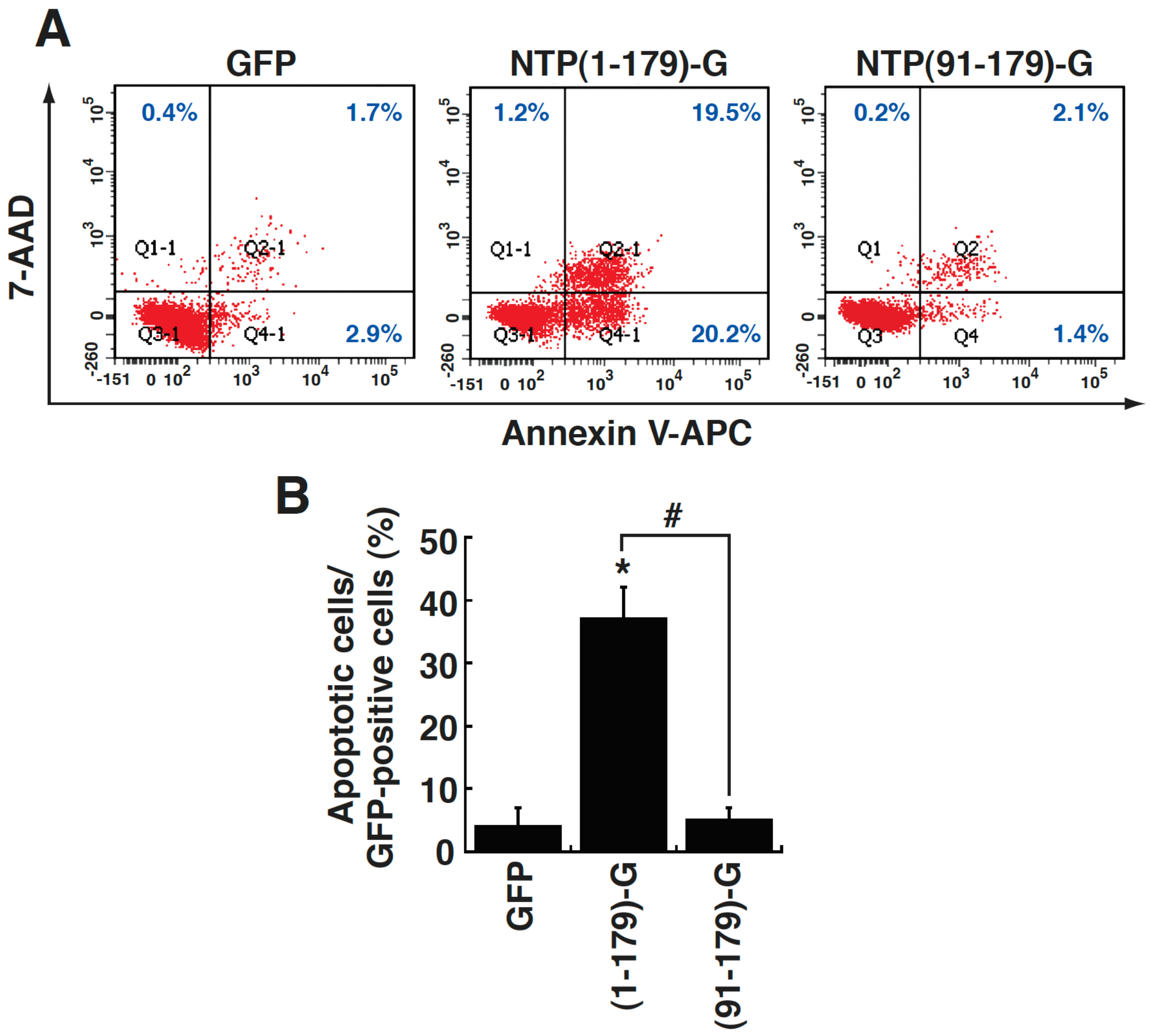

Supplementary Fig. S1. Measurement of NTPase(1-179)-GFP-mediated apoptosis by flow cytometry analysis using Annexin-V-APC and 7-AAD staining. (A) Flow cytometry profiles of transfected 293T stained with Annexin-V-APC and 7-AAD. 293T cells were transfected with the plasmid expressing GFP, NTPase(1-179)-GFP or NTPase(91-179)-GFP for 48 h. After transfection, the cells were stained with Annexin-V-APC and 7-AAD (#640930; BioLegend, San Diego, CA) and then analyzed by flow cytometry. (B) Quantification of apoptotic cells in transfected cells. The percentage of the GFP- and Annexin-V-positive cells among total GFP-positive cells was calculated. The quantitative results are plotted as mean ± SEM ($n = 3$). *, $p < 0.05$, for results compared to those from the empty vector group (GFP); #, $p < 0.05$, for results compared to those from the NTPase(1-179)-GFP-transfected group.

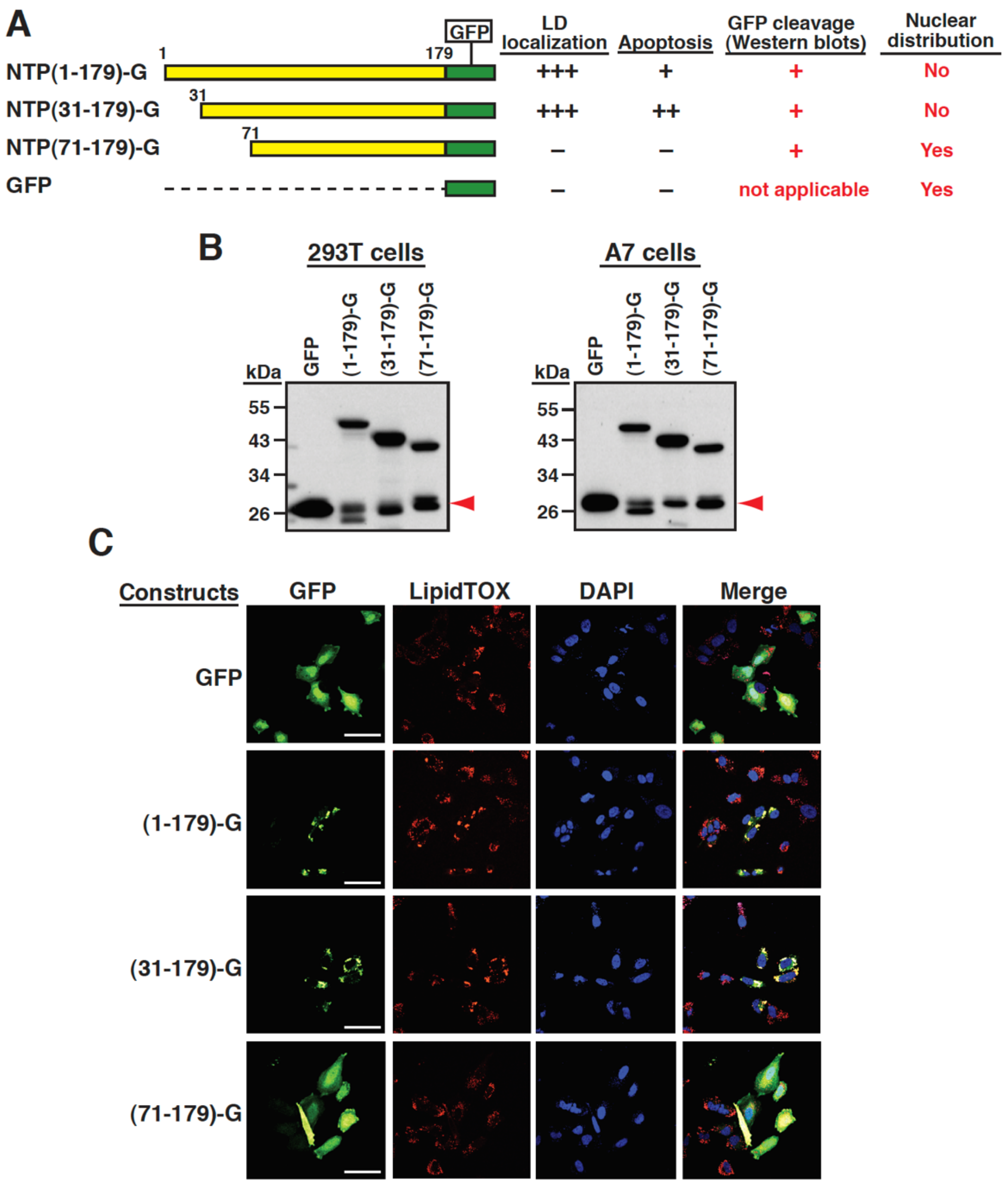

Supplementary Fig. S2. The event of the GFP cleavage from NTPase(1-179)-GFP or its deletion mutants could be observed in Western blot experiments, but not in confocal microscopy experiments. (A) Schematic diagrm of NTPase(1-179)-GFP and its deletion mutants with or without the pro-apoptotic ability. The characteristics of NTPase(1-179)-GFP and its deletion mutants are summarized in the diagram. (B) Western blot analysis showing the GFP cleavage from NTPase(1-179)-GFP or its deletion mutants in 293T or A7 cells. The red arrowheads indicate the "free GFP" bands concurrently detected in cells transfected with the NTPase(1-179)-GFP, NTPase(31-179)-GFP or NTPase(71-179)-GFP construct. The Western blotting experiments were perfomed using anti-GFP antibody (G1544; Sigma) that recognizes the N-terminal region (aa 3-17) of GFP. (C) The "free GFP" phenotype charactrized by its

diffusion into the nucleus could not be detected in NTPase(1-179)-GFP- or NTPase(31-179)-GFP-transfected cells by confocal fluorescence microscopy. Scale bars, 50 μm.

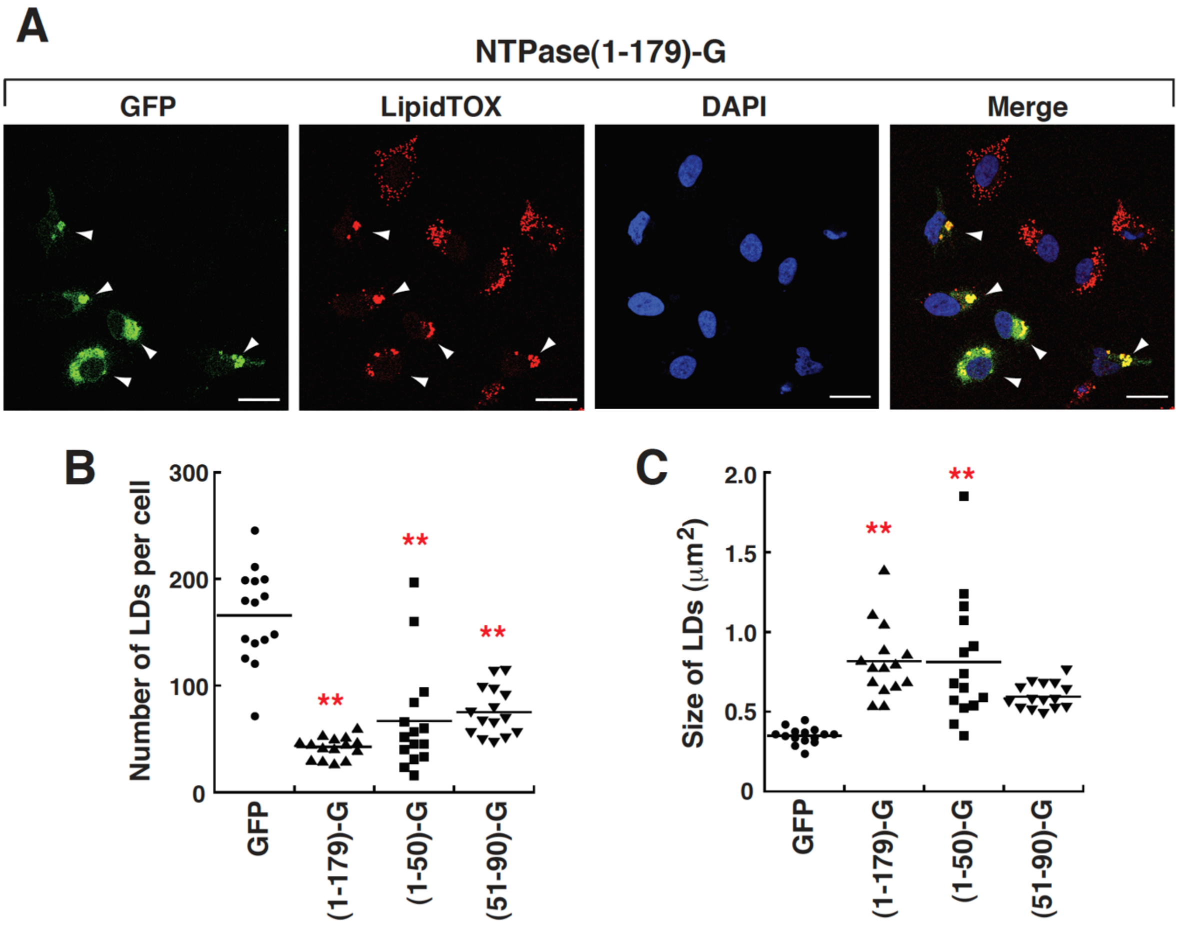

Supplementary Fig. S3. Effects of NTPase(1-179)-GFP or its deletion mutants on the number and size of lipid droplets (LDs) in transfected cells. (A) Confocal microscopic images showing the changes in the number and size of LDs in cells expressing NTPase(1-179)-GFP. The white arrowheads indicate NTPase(1-179)-GFP-positive cells. Scale bars, 20 μm. (B) Numbers of LDs in cells expressing GFP, NTPase(1-179)-GFP, NTPase(1-50)-GFP, or NTPase(51-90)-GFP. The numbers of LDs per cells were quantified by using the Image J analysis software (National Institutes of Health, Bethesda, MD, USA). $^{**}P < 0.05$ versus the control GFP group (Mann-Whitney *U* test). (C) Average sizes of LDs in cells expressing GFP, NTPase(1-179)-GFP, NTPase(1-50)-GFP, or NTPase(51-90). The LD sizes were measured by using the Image J analysis software. $^{**}P < 0.05$ versus the control GFP group (Mann-Whitney *U* test).
